# Task-sharing to support paediatric and child health service delivery in low- and middle-income countries: current practice and a scoping review of emerging opportunities

**DOI:** 10.1186/s12960-021-00637-5

**Published:** 2021-08-04

**Authors:** Yingxi Zhao, Christiane Hagel, Raymond Tweheyo, Nathanael Sirili, David Gathara, Mike English

**Affiliations:** 1 Oxford Centre for Global Health Research, Nuffield Department of Medicine, University of Oxford, S Parks Rd, Oxford, OX1 3SY UK; 2 Department of Health Policy Planning and Management, Makerere University School of Public Health, Kampala, Uganda; 3 Department of Public Health, Lira University, Lira, Uganda; 4 Department of Development Studies, School of Public Health and Social Sciences, Muhimbili University of Health and Allied Sciences, Dar es Salaam, Tanzania; 5 KEMRI-Wellcome Trust Research Programme, Nairobi, Kenya; 6 MARCH Centre, London School of Hygiene and Tropical Medicine, London, UK

**Keywords:** Paediatrics, Human resources for health, Task-shifting, Task-sharing, Clinical officer, Non-physician clinician, Clinician associate

## Abstract

**Background:**

Demographic and epidemiological changes have prompted thinking on the need to broaden the child health agenda to include care for complex and chronic conditions in the 0–19 years (paediatric) age range. Providing such services will be undermined by general and skilled paediatric workforce shortages especially in low- and middle-income countries (LMICs). In this paper, we aim to understand existing, sanctioned forms of task-sharing to support the delivery of care for more complex and chronic paediatric and child health conditions in LMICs and emerging opportunities for task-sharing. We specifically focus on conditions other than acute infectious diseases and malnutrition that are historically shifted.

**Methods:**

We (1) reviewed the Global Burden of Diseases study to understand which conditions may need to be prioritized; (2) investigated training opportunities and national policies related to task-sharing (current practice) in five purposefully selected African countries (Kenya, Uganda, Tanzania, Malawi and South Africa); and (3) summarized reported experience of task-sharing and paediatric and child health service delivery through a scoping review of research literature in LMICs published between 1990 and 2019 using MEDLINE, Embase, Global Health, PsycINFO, CINAHL and the Cochrane Library.

**Results:**

We found that while some training opportunities nominally support emerging roles for non-physician clinicians and nurses, formal scopes of practices often remain rather restricted and neither training nor policy seems well aligned with probable needs from high-burden complex and chronic conditions. From 83 studies in 24 LMICs, and aside from the historically shifted conditions, we found some evidence examining task-sharing for a small set of specific conditions (circumcision, some complex surgery, rheumatic heart diseases, epilepsy, mental health).

**Conclusion:**

As child health strategies are further redesigned to address the previously unmet needs careful strategic thinking on the development of an appropriate paediatric workforce is needed. To achieve coverage at scale countries may need to transform their paediatric workforce including possible new roles for non-physician cadres to support safe, accessible and high-quality care.

**Supplementary Information:**

The online version contains supplementary material available at 10.1186/s12960-021-00637-5.

## Background

Global strategies and initiatives for reducing child mortality and morbidity have previously focused on immunization, acute infectious diseases, and nutrition as part of the Integrated Management of Childhood Illness (IMCI) and transmission of HIV/AIDS [1, 2]. The transition to the Sustainable Development Goals (SGDs) prompted the global community to look forwards to broaden the agenda as part of “child health redesign” [3]. This includes care for complex and chronic conditions in the 0–19 years age range (referred to in this paper as paediatric and child health care) that were previously neglected and that most health systems in low- and middle-income countries (LMICs) may not be well-designed to address [3, 4].

Expanding services to encompass complex and chronic conditions is threatened by workforce shortages. The World Health Organization (WHO) estimates a gap in the supply of 18 million health workers by 2030 mostly in LMICs [5] where there is likely to be a specific challenge with the skilled paediatric workforce. Paediatrician density in 2016 was 0.5 and 6 per 100,000 children in low-income countries and lower-middle-income countries, respectively, as compared with a global mean of 32 [6]. Paediatricians also tend to work in tertiary hospitals or in the private sector, leaving few supporting primary or district-level public sector care [7]. Specialist paediatric nurses or non-physician clinicians are also scarce, and in the case of nurses legal restrictions may prevent their initiating or prescribing many forms of treatment. In most sub-Saharan African countries, general non-physician clinicians and nurses fill the gaps and deliver over 80% of primary care [6]. This leads to either de facto task-shifting or a lack of paediatric and child health care.

Task-shifting refers to “the rational redistribution of tasks among health workforce teams. Specific tasks are moved, where appropriate, from highly qualified health workers to health workers with shorter training and fewer qualifications in order to make more efficient use of the available human resources for health” [8]. Task-sharing, in comparison, emphasizes a team-based approach where different professionals work together to deliver services [9]. Task-shifting and sharing (hereinafter referred to as “task-sharing”) have a long history [10]. For child health it is implicit in IMCI strategies [11]. More recently it is embedded in care for non-communicable diseases [12], mental health [13] and children and adolescents with HIV/AIDS [14]. Informal (or unsupervised) task-sharing often occurs in rural and remote areas where mid-level clinicians and nurses perform procedures outside of their official (and sometimes legal) scopes of practice [15, 16].

In this paper, we aimed to understand existing, sanctioned forms of task-sharing and explore emerging opportunities for task-sharing to support the delivery of care for complex and chronic paediatric and child health conditions in LMICs. We conducted three parallel activities: (1) we explored which conditions have the highest disease burden for those aged 0–19 years using patterns in middle and high-income countries to indicate likely future scenarios in LMICs; (2) we investigated the training opportunities and existing policy related to task-sharing that might support expanded paediatric and child health services in five purposefully selected African countries; and (3) we conducted a scoping review of research examining task-sharing for child and adolescent health in LMICs with a specific focus on conditions other than acute infectious diseases and malnutrition that are historically shifted. Finally, we triangulated and synthesized findings to summarize the opportunities, evidence, gaps and implications for paediatric and child health service delivery in LMICs.

## Methods

### Understanding burden of diseases using the Global Burden of Disease 2019

To understand what conditions in the 0–19 years age range might need to be prioritized in LMICs we extracted disability-adjusted life-years (DALYs) of level 3 causes (diseases and injuries) for the age group “ < 20 years” in 2019 from the Global Burden of Disease study [17]. We did this for countries defined by the World Bank as: high-income, upper-middle-income, lower-middle-income, and low-income; using the patterns in the first two as an indication of likely future disease patterns in low-income and lower-middle-income countries that will occur with development. For each category of income-level, we selected the top 20 causes ranked by DALYs, and highlighted those likely to require greater emphasis in developing accessible high-quality paediatric services.

### Policy and document review of training opportunities and scope of practice

Second, we examined national training policies and professional scopes of practice in five East and Southern African countries (Kenya, Uganda, Tanzania, Malawi and South Africa). All have large gaps in the availability of skilled health professionals [18] and were the common location of research in our scoping review. We characterized the different professionals offering care, the extent of their pre-service paediatric and child health training and opportunities for post-basic training in this field. We focused on physicians, nurses and non-physician clinicians (clinical officers, clinical associates, etc.) as the cadres of interest. We searched for documents or information (e.g. from websites) from approved training institutions, relevant regulatory councils and commissions. We reviewed schemes of service, relevant acts, task-sharing policies, other broad and disease-specific national strategic plans/policies to capture their scopes of practice.

### Scoping review of research literature on task-sharing and paediatric and child health service delivery

Lastly, we conducted a scoping review [19] of studies examining the design and practice of task-sharing for paediatric and child health services in all LMICs (Additional file 1: Scoping review protocol and PRISMA diagram). In summary, we conducted a systematic search using MEDLINE, Embase, Global Health, PsycINFO, CINAHL and the Cochrane Library to identify relevant articles. We combined terms and phrases related to paediatrics, task-sharing, different cadres commonly involved in task-sharing and the Cochrane LMIC filter [20]. We included all study designs published between 1990 and 2019 in English. Table 1 shows the inclusion and exclusion criteria. After two stages of independent screening by two authors, we charted data from included papers and sorted them into three major groups based on the conditions they examined: acute infectious diseases and malnutrition; surgery (with sub-categories minor surgery, other complex surgery), emergency and intensive care; and chronic conditions (sub-categories complex and chronic conditions, mental health). For included papers we described specific health services and procedures shifted/shared, study country, study design, cadres involved, major inputs and outcomes (health worker knowledge, skill, patient outcome) as originally reported in the included papers.

**Table 1:** Inclusion and exclusion criteria for the scoping review of research evidence on task-sharing and paediatric and child health service delivery

Include	Exclude
Study objective	
• Evaluate task-sharing interventions • Report task-sharing as norm (service normally delivered by non-physician cadres)	• Use non-physician cadres but do not aim to integrate task-sharing as part of future routine care (e.g. training clinical officers to screen hearing impairment to estimate its prevalence)
Cadre	
• Clinical officer • Other non-physician clinician • Nurse • Midwife • Medical assistant	• Community health worker/volunteer • Lay health worker • Health care support staff (without professional regulation) • Patient or family
Study setting	
• Hospital • Clinics • Community only if professional involved (community nurses) In low- and middle-income countries	• Community if managed by lay health worker/community health worker • In high-income countries
Service population	
• Children and adolescent • Mixed population but state include children	• Adult • No detailed information on population
Disease and service	
• Any paediatrics preventive or curative service	• Prevention of mother-to-child transmission (PMTCT) • Emergency obstetric and newborn care • Antenatal and postnatal care • Family planning • Dental service

## Results

### Burden of disease

Focusing on those conditions not typically covered by current strategies and initiatives, Table 2 illustrates how the top-ranking conditions for which services will likely need strengthening will change as countries transition from low income to middle and high income if high-quality paediatric care is to be widely accessible. For all countries, neonatal disorders (preterm, birth asphyxia and trauma, neonatal sepsis, etc.) are the highest-ranked cause. Malaria, lower respiratory infections and diarrheal diseases are the 2nd and 3rd top-ranked causes for low-income and lower-middle-income countries, respectively, but covered by existing task-sharing strategies. Congenital birth defects are ranked 4th and 5th for low-income and lower-middle-income countries, respectively. Lower ranked but likely causes of substantial mortality and morbidity are road injuries, drowning, conflict and terrorism that require emergency and surgical care; and haemoglobinopathies and haemolytic anaemias, asthma, epilepsy and conduct disorder that are considered chronic conditions and require long-term multiple interactions with health services. Most of these conditions are also top-ranking conditions for upper-middle-income countries and high-income countries, which suggests that they will become increasingly important needs as countries develop economically.

**Table 2:**
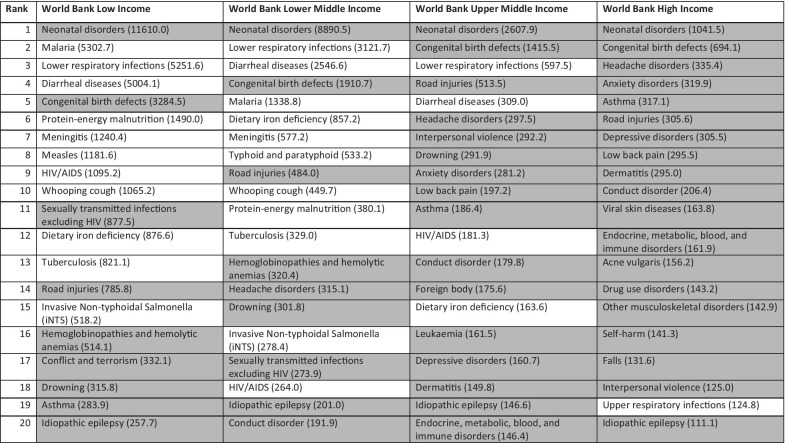
Top 20 conditions for population under 20 years ranked by DALYs in World Bank low-income, lower-middle-income, upper-middle-income and high-income countries

Conditions marked in grey are not traditionally covered by acute infectious diseases and malnutrition care

### Training opportunities and scope of practice

Table 3 summarizes the training opportunities and scope of practice related to child health for physicians, non-physician clinicians and nurses/specialist nurses in the five African countries examined. The full list (by country and by cadre) is available in the Additional File 2. This details specific opportunities for child health training, existing child health scopes of practice (where defined) and summaries of national policies and planning documents relevant to child health.

**Table 3:** Training opportunities and scope of practices related to paediatric and child health for different professional cadres in Kenya, Uganda, Tanzania, Malawi and South Africa

Cadre	Training related to child health	Other specialized training	Child health in scope of practice, relevant national policy and planning
Physicians	Most countries have 5–6 years entry-level Bachelor of Medicine and Bachelor of Surgery (MBBS) or equivalent degrees that include 3–4 months internship in paediatrics. Most countries also have Master of Medicine degree in Paediatrics and Child Health that last 2–4 years and require some working experience before entry. Some countries also offer further paediatrics sub-specialty training either through fellowship (Kenya, Uganda), Master of Science (Tanzania) or Master of Philosophy/Senior registrar (South Africa)	Master of Medicine training in major specialties including but not limited to family medicine, general surgery, internal medicine, obstetrics and gynaecology, emergency medicine that last 2–7 years and require some working experience. Similarly further sub-specialty trainings are available through fellowship, Master of Science or Master of Philosophy/Senior registrar	Scope of practice for general physicians is generally broad and findings suggest limited specific recommendations on which procedures can be performed or not. Medical specialists are allowed to carry out specialized care in their relevant field. Additionally, in some countries physicians’ responsibility include teaching and supervising students and staff (Kenya, Uganda, Tanzania), for example Kenya’s general medical officers’ duties include teaching medical and nursing students and clinical officer interns
Non-physician clinicians (Clinical officers in Kenya, Uganda, Tanzania, Malawi, assistant medical officers in Tanzania, clinical associates in South Africa)	Most countries have 3–4 years entry-level diploma or Bachelor of science degrees for non-physician clinicians which include paediatrics and child health training as an element and usually include some short internship period in paediatrics. For some countries, there are advanced diplomas in paediatrics (Kenya), child and adolescent health/paediatrics palliative care (Uganda) or post-basic Bachelor of science in paediatrics and child health (Malawi) that last 1–3 years and require some working experience before entry	Advanced diploma in other specialties that last 1.5–2 years, most commonly in family medicine, ENT, anaesthesia, ophthalmology (Kenya, Uganda, Tanzania). In Malawi there is post-basic Bachelor of Science in internal medicine, obstetrics and gynaecology, general surgery, anaesthesia and intensive care (3 years). South Africa currently offers only an honours degree in emergency medicine (1 year). Kenya also has a Master-level course for clinical officers in family medicine, emergency medicine, forensic medicine (3 years)	Scope of practice for non-physician clinicians focuses on acute infectious diseases, essential newborn care, immunization and malnutrition. Prescription of common medications is usually within the scope of practice for non-physician clinicians. Non-physician clinicians are sometimes the highest cadre in district and primary care services listed in staffing norm documents. Non-physician clinicians are usually allowed to perform minor surgery and provide emergency care as listed explicitly in their scheme of service document: e.g. Kenya’s clinical officers and Tanzania’s assistant medical officers are allowed to perform surgery per training, South Africa’s clinical associates are allowed to perform within a list of nearly 90 procedures including lumbar puncture, neonatal and paediatrics resuscitation and initiate CPAP in RDS. For chronic conditions, usually only counseling is explicitly listed in scopes of practice
Nurses and nurse specialists*	Aside from entry-level certificate/diploma/Bachelor degree in general nursing, most countries have advanced diploma in paediatrics nursing (1–2 years) and sometimes paediatrics nursing in certain speciality (neonatal nursing in Kenya and South Africa, critical care nursing in Kenya) (1.5–2 years). Malawi also has a Bachelor of Science in paediatrics nursing aside from general nursing (4 years). Master-level training in either paediatric or neonatal nursing is also common in most countries and requires a Bachelor’s degree for entry	Most countries offer advanced diplomas in nursing for other specialties, e.g. family health nursing, psychiatric or mental health nursing, palliative care nursing, critical care nursing, ophthalmic nursing (1–2 years). In Malawi there is also a Bachelor of Science in adult health nursing and community health nursing (4 years). Similarly, there is usually master-level courses in other specialties though the entry requirement for these courses includes a Bachelor’s degree	Scope of practice for nurses focuses on acute infectious diseases, essential newborn care, immunization. Prescription of essential medication is mostly not allowed for general nurses other than in Kenya where nurses are allowed to proscribe selected drugs (e.g. relating to HIV/AIDS and tuberculosis) and Malawi where nurses are allowed to prescribe at primary care level. Surgery and emergency care treatment are usually not within nurses’ scope of practice. Most countries allow nurses for mental health counseling either in task-sharing policies (Kenya, Tanzania, Malawi) or stand-alone child and adolescent mental health policies (Uganda). Malnutrition treatment is within nurses’ scope in Kenya and Tanzania. Specialist nurses usually have broader scopes of practice though rarely explicitly listed out for each different specialty

For nurses we only looked at their post-basic training opportunities excluding diploma and bachelor’s degree, despite that bachelor’s degrees could also be post-basic degrees for diploma or certificate holders

In the countries examined physician training generally lasts 5–6 years and is followed by a 1–2 years pre-licensure internship that includes some months of supervised paediatric work within a hospital. All these countries offer physicians further specialist training in paediatrics and child health and family medicine, however graduates of these specialist medical programmes are few (e.g. 20–25 pa in Kenya). It is implicit in most policies that non-specialist physicians, even if junior, are expected to provide care for chronic and complex paediatric conditions with the exception of major surgery or intensive care. As such they may be expected to supervise, teach or receive referrals from non-physician clinicians and nurses offering primary care paediatric services in the absence of specialist paediatricians.

For non-physician clinicians, most countries have 3–4-year entry-level diplomas or Bachelor’s degrees that include some elements of paediatrics and child health (mostly 3–4 short courses). These diplomas and Bachelor’s degrees also require several months of internship in paediatric wards pre-licensure with a relevant regulator. The scope of practice for non-physician clinicians usually includes prescription of common medication. In schemes of service documents some countries (Kenya, Tanzania, South Africa) also explicitly permit non-physician clinicians to perform certain typically minor surgical procedures. Three countries (Kenya, Uganda, Malawi) have advanced-level courses on paediatrics for non-physician clinicians while all countries have advanced diplomas in other relevant specialties (most commonly family medicine, anaesthesia, ophthalmology, ear nose and throat [ENT]), however this training is not specific to the paediatric age group and numbers of these specialist non-physician clinicians are much smaller than generalists.

Nurses too receive some training in child health as part of entry-level training courses and most countries offer advanced diplomas in neonatal or paediatric and child health nursing, while some also have Master’s-level training which requires a Bachelor’s degree for entry. This arrangement also applies to other relevant specialties that are not specific to paediatrics and child health (e.g. mental health/psychiatric, family medicine, critical care nursing). The scope of practice for nurses in general and often for those with advanced training is more restricted than for non-physician clinicians as in most countries such nurses are usually not authorized to prescribe. However, in primary care settings because of de facto/informal task-sharing nurses may prescribe and in some countries nurses are legally allowed to prescribe selected drugs for acute and chronic illness mostly related to HIV/AIDS and tuberculosis (Kenya) or at primary care level (Malawi). In some countries, malnutrition treatment and/or mental health counselling is within nurses’ scope of practice while in Tanzania for example, nurses are not (officially) allowed to treat severe malnutrition at health centre level.

### Scoping review on task-sharing and paediatric and child health service delivery

Table 4 shows the results of the scoping review on research evidence for task-sharing and paediatric and child health service delivery. A total of 83 papers were included for data charting, and 84% of the papers were published before 2010. The included studies covered 24 countries, 20 of which were African, most commonly Malawi (*n* = 14), Kenya (*n* = 12), Uganda (*n* = 12), South Africa (*n* = 8) and Tanzania (*n* = 6). Forty-nine studies assessed task-sharing as a new intervention, and 34 studies reported task-sharing as a norm, i.e. mentioned that services were routinely delivered by non-physicians but the study aim was not assessing task-sharing. Sixty-five studies used quantitative approaches (cross-sectional (*n* = 25), before–after (*n* = 13), non-randomized trials (*n* = 9)). Ten used qualitative approaches either interviews (*n* = 5), case study/review (*n* = 5), mostly investigating how task-sharing initiatives were implemented and health workers’ perspectives. Another 8 studies used mixed-method approaches. For the outcomes of care that were being shared assessed (*n* = 73 quantitative and mixed-method studies), 18 studies assessed the lower cadres’ knowledge, 28 studies their skills, and 35 patient outcomes including mortality, length of hospital stay, follow-up and adherence rates and patient satisfaction. We now consider findings organized by the type and complexity of conditions.

**Table 4:** Research evidence on task-sharing and paediatric and child health service delivery: finding from a systematic search and scoping review

Author	Service shifted/shared	Country	Study type	Sharing from/to	Input	Outcome
**Minor surgery**						
Bowa et al. 2013 [21]	Neonatal male circumcision	Zambia	Non-randomized trial	From: Doctor/specialist To: Doctor, NW, CO, nurse	Didactic lectures, practice on models of neonatal genitalia and clinical practice	Total adverse event rate 4.9% though including performed by physicians
Kankaka et al. 2017a [22]	Early infant male circumcision	Uganda	Non-randomized trial	From: Doctor/specialist To: CO, NW	5-day didactic training, hands-on surgical training on 15 cases	Knowledge and competency score increased for CO, NW Pain scores similar in two groups, adverse event rate 3.5%
Kankaka et al. 2017b [23]	Early infant male circumcision	Uganda	RCT	From: Doctor/specialist To: CO, NW	Trained (no detail of training)	Adverse event rate 2.4% for CO and 1.6% for NW, maternal satisfaction high (99.6% and 100%, respectively)
Young et al. 2012 [24]	Early infant male circumcision	Kenya	Non-comparative evaluation	From: Doctor/specialist To: CO and nurse	Not reported	Adverse event rate 2.7% and patient satisfaction rate 96%
Frajzyngier et al. 2014 [25]	Male circumcision	Kenya	Non-randomized trial	From: Doctor/specialist To: CO, nurse	Training developed based on WHO/UNAIDS manual	Adverse event rate (2.1% for nurses and 1.9% for CP) and client satisfaction over 99%
Mwandi et al. 2012 [26]	Male circumcision	Kenya	Non-comparative evaluation	From: Doctor/specialist To: CO, Nurse	Not reported	Adverse event rate 1.4% for CO and nurse, respectively, and 0% for medical officer
Alawamlh 2019 [27]	Male circumcision	Kenya	RCT	From: Doctor/specialist To: NPC	Not reported	Mean pain score, mean operation time and rate of complete wound healing similar in two RCT arms, no adverse event
Rode et al. 2015 [28]	Burn service (minor)	South Africa	Case study/review	From: Doctor/specialist To: Doctor, nurse	Referral to higher level facility	Not reported
**Other complex surgery and intensive care**						
Tyson et al. 2014 [29]	Burn surgery, neurosurgery (VP shunting), general surgery ENT surgery,	Malawi	Non-randomized trial	From: Specialist To: CO	3-year education and 1-year rotation clinical internship Oversight and supervision	Higher re-operation rate (7.1% for doctors, 17% for CO), similar complication rate (4.5% vs. 4.0%), mortality rate (2.5% vs. 2.1%), length of stay (10 vs. 24 day) considering case mix (burn usually managed by COs)
Wilhelm et al. 2011 [33]	VP shunting	Malawi	Non-randomized trial	From: Specialist To: CO	3-year pre-service training, 1-year internship Study compared effect with and without supervision	Postoperative mortality rates (6.6% vs 5.9%), wound infection rates (3.3% vs 3.9%), rates of early shunt revision (0 vs. 3.9%) in CO only and surgeon present group. Length of stay shorter in surgeon present group
Tindall et al. 2005 [30]	Clubfoot deformity	Malawi	Non-comparative evaluation	From: Doctor/specialist To: CO	3-day residential and practical workshop 1:1 teaching & supervision	98 of 100 clubfeet in our study were corrected to plantigrade or better by COs
Wilhelm et al. 2017 [31]	Major amputation, open reduction, internal fixation with plates	Malawi	Non-randomized trial	From: Specialist To: CO	Diploma in clinical orthopaedics (18 months)	Peri-operative mortality 15.6% vs 12.9%, blood transfusion 32.5% vs. 41.9%, infection 16.9% vs. 19.4%, re-operation 15.6% vs. 19.4%, length of stay 18d vs 20d in CO only and surgeon present group
Grimes et al. 2014 [32]	Amputation, fracture, etc.	Malawi	Cost-effectiveness	From: Doctor/specialist To: CO	Not reported	Cost-effectiveness of providing orthopaedic care through CO training was US$92.06 per DALY averted
**Emergency care**						
Tiemeier et al. 2013 [35]	Emergency medicine	Uganda	Cross-sectional	From: Doctor/specialist To: NPC	Not reported	Not reported
Chamberlain et al. 2015; Rice et al. 2016 [36, 37]	Emergency medicine	Uganda	Before-after, Non-comparative evaluation	From: Specialist To: Emergency care practitioner (nurse, new cadre)	Initially paired with emergency medicine physician for nine months, continued teaching by rotating volunteer physicians	3-day in-hospital mortality rate 5.04% for unsupervised, 2.90% for supervised. Patients that not severely ill mortality rate showed no difference (2.17% vs. 3.09%) Under-five case fatality rate 1.9% for malaria, 4.1% for pneumonia, 1.6% for trauma and 6.8% for malnutrition
Olayo et al. 2019 [34]	CPAP	Kenya	Non-comparative evaluation	From: Specialist To: Doctor, nurse, CO	2-day training session	Knowledge and skills scores higher for trained providers Total mortality rate 24%, 95% no adverse event
James et al. 2019 [38]	Trauma and ETAT	Ghana	Before–after	From: Doctor/specialist To: Physician assistant, nurse, midwife	ETAT + course and one module of trauma teaching	Confidence and knowledge score increased for injury management after training
**Complex and chronic conditions**						
Aliku et al. 2018 [45]	RHD prevention and management	Uganda	Before–after study	From: Doctor/specialist To: CO, nurse, nurse assistant, midwife	3-month RHD education training programme	Knowledge score improved BPG adherence level remained similar (95.8% vs 94.5), no adverse event following decentralization
Sanyahumbi, 2019 [46]	RHD management	Malawi	Before–after study	From: Doctor/specialist To: Doctor, nurses, CO	3 half-day workshop	Improvement in knowledge score, more comfortable prescribing/injecting benzathine penicillin
Sims et al. 2015 [39]	RHD screening	Malawi	Cross-sectional	From: Specialist To: CO	3 half-day didactic & computer-based training, 2-day clinical attachment	Kappa between specialist and CO was 0.72; overall sensitivity 0.92, specificity 0.80
Sims Sanyahumbi et al. 2017 [40]	RHD screening	Malawi	Cross-sectional	From: Specialist To: CO	3 half-days didactic & computer-based training, 2 h practical learning	Mean kappa statistic comparing CO with paediatric cardiologist was 0.72; sensitivity 0.91, specificity 0.65
Beaton et al. 2016 [41]	RHD screening	Brazil	Cross-sectional	From: Doctor/specialist To: Nurse, technician	Standardized, computer-based training	Sensitivity and specificity 85% and 87%
Engelman et al. 2015 [42]	RHD screening	Fiji	Cross-sectional	From: Doctor/specialist To: Nurse	Classroom training for one-week, practical session	Knowledge score increased, 98% nurses of adequate quality for diagnosis
Colquhoun et al. 2013 [43]	RHD screening	Fiji	Cross-sectional	From: Doctor/specialist To: Nurse	A week-long training workshop, 2 weeks of screening under supervision 11-step basic algorithm	Sensitivity of 100% and 83%, and a specificity of 67.4% and 79%, respectively, for the two nurses
Ploutz et al. 2016 [44]	RHD screening	Uganda	Cross-sectional	From: Doctor/specialist To: Nurse	4-h didactic, case study & computer-based training, 2-day hands-on session	Sensitivity of 74.4%, specificity of 78.8%
Eberly et al. 2018 [70]	Heart failure screening and treatment	Rwanda	Cross-sectional	From: Specialist To: Nurse	Not reported	Nurse-performed echocardiography had sensitivity and specificity of 81% and 91% for other RHD;
Patel et al. 2019 [71]	Epilepsy diagnosis and management	Zambia	Before–after study	From: Doctor/specialist To: CO	3-week six training model and open case discussion	Increased knowledge on epilepsy medication management, recognition of focal seizure, etc.; limited knowledge on provoked seizures, diagnostic studies, general aetiologies
Harris and Harris 2013 [47]	Epilepsy treatment	Uganda	Case study/review	From: Specialist To: CO	Extra training in epilepsy	Higher patient follow-up (70%) in satellite clinics as compared with hospitals, better seizure management
Kengne et al. 2008 [48]	Epilepsy treatment	Cameroon	Case study/review	From: Doctor/specialist To: Nurse	Physician available as needed Dosage chart and protocol	Total mortality rate 2.7% and reduced seizure during follow-up period
Abbo et al. 2019 [50]	Epilepsy treatment	Uganda	Case study/review	From: Doctor/specialist To: CO, nurse, others	Not reported	Not reported
Some et al. 2016 [49]	Epilepsy management, sickle cell	Kenya	Non-comparative evaluation	From: CO To: Nurse	1-week didactic & clinical case scenario Supervising CO Structured clinical support tool	Adherence to protocol for epilepsy: patient consultation (82%), weight checked (55%)
Paiva et al. 2012 [72]	CNS tumour	Brazil	Case study/review	From: Doctor/specialist To: Nurse specialist	Not reported	Not reported
Kengne, Sobngwi, et al. 2008 [73]	Asthma diagnosis and treatment	Cameroon	Non-randomized trial	From: Doctor/specialist To: Nurse	4-day training, refresher course 1 year later Physician available as needed Clinical management algorithm	Median follow-up 2 visits, 39.1% re-hospitalization rate, no death in child and adolescent group
Buser, 2017 [74]	Haematology service	Tanzania	Case study/review	From: Doctor/specialist To: Nurse	2-week collaborative education programme training	Not reported
Mafwiri et al. 2014 [75]	Eye care prophylaxis, ocular conditions control	Tanzania	Before–after study, interview	From: Doctor/specialist To: CO, nurses, students	Training, educational materials Referral and torch for examination	Better knowledge on eye conditions and diagnostics skills Better management (referral) of cataract and trauma
**Mental health**						
Rossouw et al. 2016, 2018; van de Water et al. 2017, 2018 [51–54]	Counselling for PTSD	South Africa	RCT, interview	From: Specialist To: Nurse	1-year advanced psychiatry diploma, 4-day workshop, 16-h practical training Group supervision every week	Improved patient PTSD (interviewer-rated from 35.32 to 9.29 at 6 month), depression (from 31.4 to 10.12), global functioning (from 52.01 to 67.26)
Tesfaye et al. 2014 [55]	Child psychiatry	Ethiopia	Case study/review	From: Doctor/specialist To: Non-physician clinician	2-week training course and 4-week internship	Improved confidence in caring for child patient
Akol et al. 2017 [56]	Mental, neurological, substance use disorder identification	Uganda	Before–after study	From: Doctor/specialist To: CO, nurse, midwife	5-day residential training including classroom and practicum	Improvement in mean test score for mental health knowledge, clinical officers had a higher mean score

*RCT* randomized controlled trial; *CO* clinical officer; *NW* nurse and midwife

#### Acute infectious diseases and malnutrition

Forty-four papers examined acute infectious diseases and malnutrition, mostly examining HIV/AIDS testing, antiretroviral therapy (ART), and neonatal disorders as addressed in IM(N)CI and Emergency Triage Assessment and Treatment (ETAT). As we are more concerned with other conditions we do not present their findings here, but detailed characteristics of these studies are presented in Additional File 1.

#### Minor surgery

Seven studies reported male circumcision for infants or adolescents performed by clinical officers, nurses and midwives in Kenya, Uganda and Zambia [21–27]. This is a highly specific “acute” service focusing on HIV/AIDS prevention that does not generally extend the professional role too far and only requires short training (e.g. 5 days didactic and hands-on training [22]) with limited need for ongoing supervision. Studies report a relatively low adverse event rate (from 0% [27] to 4.9% highest [21]) and high patient and/or maternal satisfaction rate [23, 24]. One study reported minor burn services (wound care) provided by nurses at primary care while major burns were referred to secondary hospitals [28].

#### Other complex surgery

Five studies reported on amputation for some complex fractures, clubfoot corrective surgery, other orthopaedic surgery, burn surgery, ENT surgery and ventriculo-peritoneal (VP) shunting [29–33]. Three of these examined orthopaedic surgery delivered by clinical officers in Malawi and they reported an acceptable mortality rate when performed unsupervised as compared with specialists [31] and high cost-effectiveness [32]. One non-randomized trial in Malawi suggested that when working together in central hospitals different cases were shared between clinical officers and physicians: most burn surgery, foreign body removal cases and ventriculo-peritoneal (VP) shunt placement were performed by clinical officers whereas general surgery, urology and congenital cases were more often performed by physicians, both groups had similar mortality and complication rates [29]. Another study focusing on VP-shunting in Malawi suggested that clinical officers operating alone had a slighter higher mortality rate than with a surgeon present (6.6% vs. 5.9%), but comparable infection and shunting revision rates [33].

#### Emergency care

Five studies reported on “emergency care” in Kenya, Uganda and Ghana [34–38]. Task-sharing for emergency care usually includes additional in-service training to build on non-physician clinicians and nurses’ pre-service training and requires initial pairing with specialists. In one Ugandan study, nurses were trained for 2 years as emergency care providers (a new cadre) with the goal that they could perform assessment, diagnosis and initiate treatment independently without physician supervision. However, the mortality rate nearly doubled when they practised unsupervised (5.04%) vs. supervised (2.90%), though for patients that were not severely ill there was no significant difference in mortality rate (3.09% vs. 2.17%) [36, 37]. One study also examined continuous positive airway pressure for neonatal and paediatric patients in Kenya [34] and reported an overall 24% mortality rate when performed by nurses and clinical officers. The other two studies reported only an increase in health worker knowledge of those taking on a new task [35, 38].

#### Complex and chronic conditions

Eighteen studies examined care for rheumatic heart diseases (RHD), epilepsy, sickle cell, asthma, eye care and tumours across seven African countries, Brazil and Fiji. Six studies examined the shifting of RHD screening to clinical officers, nurses, midwives and other cadres. With several days of additional training, these cadres achieved substantial agreement rates in RHD diagnosis using echocardiography as compared with specialists [39–44]. Two studies further reported on RHD treatment where health worker knowledge increased after training [45, 46]. One reported good patient adherence rates for monthly prophylaxis after initial diagnosis and treatment at referral hospitals followed by task-shifting to health workers in local clinics [45]. Five studies investigated epilepsy. Diagnosis and management by clinical officers and nurses achieved better patient follow-up [47] and patient outcomes, e.g. mortality rate and seizure incidence [48] when care was decentralized rather than centralized in hospitals. In a study in Kenya, epilepsy treatment was shared from clinical officers to nurses who received additional training, dosage and management charts and continuous on-site supervision from clinical officers. Nurses showed moderate adherence to treatment protocols [49]. However, a qualitative study in Uganda showed that clinical officers and nurses in primary care had inadequate supervision and multidisciplinary rehabilitation team support when providing epilepsy care and they gradually lost their skills [50].

#### Mental health

Of six studies four were from one set of work in South Africa. These included randomized controlled trials of two different post-traumatic stress disorder (PTSD) treatments delivered by nurses for adolescents with subclinical PTSD in schools accompanied by qualitative work [51–54]. After initial diagnosis by a psychiatric nurse and/or a clinical psychologist, patients received treatment from nurses who were completing a 1-year advanced psychiatry diploma. Nurses also received group supervision every week from one clinical psychologist. Task-shifting in this study achieved satisfactory health outcomes (improved patient’s PTSD score, depression and global functioning [51, 52]) and was well-accepted by patients and nurses despite the latter initially resisting supervision [53, 54]. Two other studies in Ethiopia [55] and Uganda [56], respectively, reported that health worker knowledge and skills improved after training for child and adolescent mental health.

## Discussion

In this review, we explore for paediatric and child health services in LMICs likely areas of considerable service need. We focus on current approaches to training non-physicians and nurses to support such care in five African countries and summarize existing findings from research on task-sharing for provision of complex and chronic paediatric and child health conditions. We discuss below the implications, potential opportunities and research gaps in work on task-sharing and paediatric and child health service delivery.

### Task-sharing for paediatric surgery, emergency and intensive care

We found some training opportunities but rather limited policy opportunities for surgery, emergency and intensive care task-sharing. Non-physician clinicians could receive post-basic training in surgery, anaesthesia and emergency medicine. While most previous research evidence on task-sharing to non-physician clinicians or nurses focuses on adult and obstetrics services [57], research evidence on task-sharing for paediatric surgery has emerged over the past decade on circumcision, burn surgery, orthopaedics and VP shunts. Surgery for more complex cases (e.g. congenital defects) seems restricted to the few trained physicians despite a high disease burden. Similarly, while there are advanced courses on critical care nursing and reasonably well-established short-courses for emergencies, e.g. ETAT/ETAT+ and helping babies breathe (HBB), these short courses do not aim to formally establish new professional roles or expand scopes of independent practice. In the few studies that are done on sharing complex surgery or emergency care the mortality rate of patients managed by unsupervised clinical officers and nurses may be higher compared with patients managed by physicians or supervised clinical officers/nurses. Given the general deficits in the medical workforce especially in paediatric surgery and emergency care specialists [6, 58], it would seem worth exploring a more deliberate effort to develop specific paediatric task-sharing roles at hospital-level as has been practised for adults in Tanzania’s assistant medical officers [59].

### Task-sharing for paediatric chronic conditions

Task-sharing for these chronic conditions is likely to occur frequently in primary care to non-physician clinicians and nurses due to the shortage of physicians at this level [6]. Nonetheless, this is not clearly reflected in their training curricula and scopes of practices. Despite some examples of advanced paediatrics and family medicine training that covered most paediatrics subspecialties, the production of such professions is relatively small. For example, in 2018 there were only 255 clinical officers and 119 nurses with higher diplomas or master-level paediatric qualifications in Kenya despite some of these courses being introduced in the late 1970s [7, 60, 61].

Research evidence on task-sharing for chronic conditions is limited. Studies focus on mental health, RHD and epilepsy. Most were reasonably small in scale and examined either focused initial diagnosis (echocardiography for RHD diagnosis), or follow-up treatment in lower-level health facilities provided by clinical officers or nurses alone. The implementation experiences reported for mental health and epilepsy treatment suggest successful task-sharing requires sustained training and supervision, uninterrupted supplies of medications and sometimes support from specialized teams to meet complex medical and rehabilitation needs [50, 53]. The challenges posed are similar to those for other non-communicable diseases and with the potential need for regular, scheduled follow-up countries need to consider how best to deliver this together with effective linkages between system levels.

### Implications and future considerations

Countries with very few specialists in paediatrics or family medicine and that rely on these cadres to extend access to paediatric and child health care for more complex and chronic conditions might take decades to achieve this given the challenges of training capacity, duration and cost. Task-sharing to cadres with shorter training could be one solution to this human resources gap. However, several issues need to be highlighted. Providing such paediatric and child health services requires a system-approach with integrated models of care spanning healthcare organizations, communities, patients, and sometimes other stakeholders [62]. For example, long-term disability requires sustained interactions with the medical and rehabilitative services [4, 50, 63]. Careful, strategic thinking on the mix of cadres, their roles, regulation, financing and training and supervision and management of teams and services are needed [64–66]. To inform this much more might be learned from better evaluation of existing experience. Governments, regulatory councils and training institutions also need to enable changes in education, legislation, policy and financing well in advance of future expansion of service scope and scale as producing the desired mix of professions and skills may take years or even decades [64, 65].

Task-sharing strategies should also be mindful of professional identities and hierarchy [66, 67]. If further sharing of what are traditionally medical doctors and specialists’ professional responsibilities with other cadres is being considered, policy-makers need to win doctors’ endorsement and support to ensure effective task-sharing and the supportive supervision and team work that is needed for quality care. The planning needs to be context-specific, based on countries’ existing structures, available resources, previous experiences of task-sharing and future planning for universal health coverage. There are multiple specific examples of more specialist roles for non-physician clinicians and nurses with post-basic training in paediatrics and child health. However, graduates of such programmes are relatively few and it is not clear that their development is part of broader strategic and holistic thinking of how paediatrics and child health care services might be delivered at scale by teams possibly comprising multiple professions. For example, legal restrictions on nurses’ or non-physicians’ prescribing even after specialist training may undermine efforts to expand coverage. To this end, better research is needed on the outcomes, quality of care and costs associated with task-sharing if it is to be a means of improving coverage and quality of care rather than associated with the provision of “second-rate” services [68, 69].

### Limitations

Our study is not without limitations. Due to data and resource availability, we present secondary data on disease burden for 2019 instead of predicting the DALYs for the future. For the training opportunities and scope of practice review, we only examined five East and Southern Anglophone African countries. Paediatrician density is lowest in sub-Saharan Africa [6] and non-physician clinicians are more common in this region as evidenced by the fact that most identified research was from these countries. For the scoping review, we are only able to search and synthesize evidence reported in the research literature, in some circumstances task-sharing may already happen and become the norm, and therefore may not be reported in research papers. We also focused exclusively on task-sharing to professionals in the health sectors although it is well-known that other carers play a huge role in service delivery for chronic conditions.

## Conclusion

The child health redesign agenda provides an ambitious outlook for children and adolescents in the SDG era, however addressing the human resources gap is a key challenge to further expand service provision. Our review summarized the current practices and emerging opportunities for task-sharing to support paediatric and child health service delivery in LMICs. While training opportunities for expanded services exist they produce relatively small numbers and non-physician clinicians’ and nurses’ training opportunities and scopes of practice are rather restricted. Aside from the historically shifted care of acute infectious diseases and malnutrition, there is limited research evidence on outcomes and quality of care for other forms of task-sharing. Service delivery arrangements for other priority conditions (congenital anomalies, major injuries, other chronic conditions, e.g. cancers, haemoglobinopathies) should be the subject of future research. To achieve coverage at scale countries may need to transform their paediatric and child health workforce including possible new roles for nurses, non-physician clinicians and other allied health workers to support safe, accessible and high-quality care.

## Supplementary Information

**Additional file 1:** Scoping review appendix.

**Additional file 2:** Training opportunities and scope of practices related to child health for mid-level health workers in Kenya, Uganda, Tanzania, Malawi and South Africa.

## Data Availability

All data relevant to the study are included in the article or uploaded as additional files.

## Abbreviations

ART: Antiretroviral therapy
DALYs: Disability-adjusted life-years
ETAT: Emergency Triage Assessment and Treatment
IMCI: Integrated Management of Childhood Illness
PTSD: Post-traumatic stress disorder
LMICs: Low- and middle-income countries
RHD: Rheumatic heart diseases
VP: Ventriculo-peritoneal
WHO: World Health Organization
