# Supplementary material for: Task-sharing to support paediatric and child health service delivery in low- and middle-income countries: current practice and a scoping review of emerging opportunities
Source: Hum Resour Health. 2021 Aug 4;19:95. doi: 10.1186/s12960-021-00637-5 (PMC8336272; doi:10.1186/s12960-021-00637-5)
Supplement: Supplementary file 1 — Additional file 1: Scoping review appendix. [file 12960_2021_637_MOESM1_ESM.docx]

## Appendix 1. Scoping review appendix

We followed the five steps of Arksey and O’Malley method (1) for scoping review to identify the research evidence on task shifting/sharing for child and adolescent health.

### Identifying the initial research questions

To guide the search strategy and ensure that a broad range of literature was captured, our research question was: “What are the design and practice of task shifting/sharing for child and adolescent health in low- and middle-income countries (LMICs)?”. We are specifically interested in besides acute care (e.g. as targeted in Integrated Management of Childhood Illness, IMCIs, HIV/AIDS), how task shifting/sharing may support an expanded set of services as these are redesigned to meet the needs of previously neglected age or illness groups. We defined task shifting in line with WHO as “the rational redistribution of tasks among health workforce teams. Specific tasks are moved, where appropriate, from highly qualified health workers to health workers with shorter training and fewer qualifications in order to make more efficient use of the available human resources for health” (2). We defined child and adolescent as population aged between 0 and 19 also align with WHO (3).

### Identifying relevant studies

In consultation with an experienced librarian, we conducted a systematic search using MEDLINE, Embase, Global Health, PsycINFO, CINAHL and the Cochrane Library to obtain relevant articles. We included all kinds of study design published between 1990-2019 in English only due to time and resource constraints. We combined keyword terms and phrases related to child and adolescent (infant, newborn, paediatrics, adolescent, etc.), task shifting/sharing and different cadres commonly involved in task shifting/sharing (substitution, skill mix, advanced practice nurse, specialist nurse, non-physician clinician, clinical officer, assistant medical officer, etc.) and the Cochrane low- and middle-income filter (4). See eTable 1 for the example search strategy in Embase.

### Study selection

We included studies if they examined task-shifting/sharing of any preventive or curative service for conditions for children and adolescent, either as norm or as task shifting/sharing focused interventions; conducted in a health facility setting (district hospital, referral hospital, regional hospital or central hospital), or in primary care facility (health centre and clinic), or by professionals in community settings (e.g. nurses working in schools for treating post-traumatic stress disorder [PTSD]); in low- and middle-income countries.

We excluded studies if they examined task shifting/sharing of any service not primarily for children and adolescent (e.g. PMTCT, obstetrics and delivery related, or adult only); dental services; performed completely by community health workers, lay health workers or patients (The reason for such exclusion is that there is already abundant evidence on CHWs and out of scope for our analysis); and care shifted to private pharmacies; task shifting/sharing that falls within the domain of specific professional training (e.g. a trainee paediatrician or surgeon taking on tasks of a fully qualified professional); used new cadres to provide any service as part of an intervention but the primary focus of the study is not task shifting/sharing and did not aim to integrate task shifting/sharing as part of future routine care (e.g. training CO to screen hearing impairment to estimate its prevalence). We also excluded systematic reviews at the full-text stage but their references were checked for additional studies.

While we included services for acute care in our search and selection (HIV/AIDS, malaria, tuberculosis, malnutrition, and other acute care targeted in IMCIs etc.), we did not examine them in detail.

After deduplication, we imported the citations into Abstrackr for initial title and abstract screening (5). YZ reviewed all the titles and abstracts to assess eligibility for full-text review, and a random subset of 40% were reviewed by CH. We used Gwet’s AC1 to assess agreement rate between reviewers, which is an agreement coefficient that perform better than Cohen’s Kappa when prevalence is low (6,7). The agreement rate on which study to include between YZ and CH for this subset was high (percent agreement 0.94 [excellent], Cohen’s Kappa 0.28 [fair], Gwet’s AC1 0.93 [excellent]) therefore we proceeded with the full-text review. YZ reviewed all full texts as primary reviewer and CH acted as secondary reviewer to determine inclusion (percent agreement 0.95 [excellent], Cohen’s Kappa 0.90 [substantial], Gwet’s AC1 0.91 [substantial]). We resolved disagreements on inclusion at title and abstract stage or at full-text stage by discussion among the two reviewers. The PRISMA flow diagram is shown in eFigure 1.

### Data charting and collation

YZ charted data from included articles and entered them into a Microsoft Excel spreadsheet. We extracted the following data items: author(s), year of publication, country of study, study setting, study population, study aim, study design, task shifting from, task shifting to, whether task shifting mentioned as norm or new intervention, diseases and conditions (categorized using the Global Burden of Diseases level 3 causes), specific service shifted, input, outcome (knowledge, skill, patient outcome), and other implementation experiences.

### Summarising and reporting findings

While for the current review we are interested in describing the task-shifting practices, focusing on the following aspects:

- What services are task shifted/shared? From what cadre to what cadre?
- What additional input are needed for such task shifting/sharing to happen? (e.g. training, support, supervision, tools)
- What are the outcome variables the quantitative studies included for evaluation or comparison? (e.g. health worker knowledge, and skills, patient related outcomes)
- What are the reported experiences and implementation challenges in qualitative studies?

We categorized the paper using the diseases and conditions information into three major groups: (A) historically shifted, which refers to acute medical care for neonates as covered in IMCI, HIV/AIDS, malaria, tuberculosis; (B) conditions that require emergency, intensive care or surgery; and (C) chronic health conditions. Our reporting mostly focuses on the latter two groups. We described within our included research literature, what are the specific health services and procedures shifted/shared, the study country, the study design, the cadres involved, the major inputs and the patient outcomes as reported in the paper.

### eTable 1. Example search strategy in Ovid_Embase

Database(s): Embase 1974 to present
Search Strategy:

| **#** | **Searches** | **Results** |
| --- | --- | --- |
| 1 | (infan* or newborn* or new-born* or neonat* or baby* or babies or toddler* or minor* or boy* or girl* or kid or kids or child* or schoolchild* or adolescen* or juvenil* or youth* or teen* or pubescen* or pediatric* or paediatric* or peadiatric* or school* or prematur* or preterm* or "pre term*" or prepub* or pre-pub* or preschool* or pre-school* or kindergarten* or nursery* or preadolescen* or pre-adolescen*).tw. | 3559535 |
| 2 | adolescent/ or exp child/ or exp infant/ | 3327156 |
| 3 | 1 or 2 | 4768674 |
| 4 | (task* adj2 (shift* or shar*)).ti,ab. | 3513 |
| 5 | (delegation or substitution or "skill mix*").ti,ab. | 153129 |
| 6 | ("nurse practitioner*" or "advanced practice nurs*" or "nurse clinician*" or "specialist nurs*" or "nurs* specialist*").ti,ab. | 28132 |
| 7 | ("non-physician clinician*" or "nonphysician clinician" or "clinical officer*").ti,ab. | 546 |
| 8 | ("physician assistant*" or "assistant medical officer*" or "clinical assistant*").ti,ab. | 4865 |
| 9 | 4 or 5 or 6 or 7 or 8 | 187833 |
| 10 | 3 and 9 | 24666 |
| 11 | (afghanistan or albania or algeria or american samoa or angola or "antigua and barbuda" or antigua or barbuda or argentina or armenia or armenian or aruba or azerbaijan or bahrain or bangladesh or barbados or republic of belarus or belarus or byelarus or belorussia or byelorussian or belize or british honduras or benin or dahomey or bhutan or bolivia or "bosnia and herzegovina" or bosnia or herzegovina or botswana or bechuanaland or brazil or brasil or bulgaria or burkina faso or burkina fasso or upper volta or burundi or urundi or cabo verde or cape verde or cambodia or kampuchea or khmer republic or cameroon or cameron or cameroun or central african republic or ubangi shari or chad or chile or china or colombia or comoros or comoro islands or iles comores or mayotte or democratic republic of the congo or democratic republic congo or congo or zaire or costa rica or "cote d’ivoire" or "cote d’ ivoire" or cote divoire or cote d ivoire or ivory coast or croatia or cuba or cyprus or czech republic or czechoslovakia or djibouti or french somaliland or dominica or dominican republic or ecuador or egypt or united arab republic or el salvador or equatorial guinea or spanish guinea or eritrea or estonia or eswatini or swaziland or ethiopia or fiji or gabon or gabonese republic or gambia or "georgia (republic)" or georgian or ghana or gold coast or gibraltar or greece or grenada or guam or guatemala or guinea or guinea bissau or guyana or british guiana or haiti or hispaniola or honduras or hungary or india or indonesia or timor or iran or iraq or isle of man or jamaica or jordan or kazakhstan or kazakh or kenya or "democratic people’s republic of korea" or republic of korea or north korea or south korea or korea or kosovo or kyrgyzstan or kirghizia or kirgizstan or kyrgyz republic or kirghiz or laos or lao pdr or "lao people's democratic republic" or latvia or lebanon or lebanese republic or lesotho or basutoland or liberia or libya or libyan arab jamahiriya or lithuania or macau or macao or "macedonia (republic)" or macedonia or madagascar or malagasy republic or malawi or nyasaland or malaysia or malay federation or malaya federation or maldives or indian ocean islands or indian ocean or mali or malta or micronesia or federated states of micronesia or kiribati or marshall islands or nauru or northern mariana islands or palau or tuvalu or mauritania or mauritius or mexico or moldova or moldovian or mongolia or montenegro or "montenegro (republic)" or morocco or ifni or mozambique or portuguese east africa or myanmar or burma or namibia or nepal or netherlands antilles or nicaragua or niger or nigeria or oman or muscat or pakistan or panama or papua new guinea or new guinea or paraguay or peru or philippines or philipines or phillipines or phillippines or poland or "polish people's republic" or portugal or portuguese republic or puerto rico or romania or russia or russian federation or ussr or soviet union or union of soviet socialist republics or rwanda or ruanda or samoa or pacific islands or polynesia or samoan islands or navigator island or navigator islands or "sao tome and principe" or saudi arabia or senegal or serbia or seychelles or sierra leone or slovakia or slovak republic or slovenia or melanesia or solomon island or solomon islands or norfolk island or norfolk islands or somalia or south africa or south sudan or sri lanka or ceylon or "saint kitts and nevis" or "st. kitts and nevis" or saint lucia or "st. lucia" or "saint vincent and the grenadines" or saint vincent or "st. vincent" or grenadines or sudan or suriname or surinam or dutch guiana or netherlands guiana or syria or syrian arab republic or tajikistan or tadjikistan or tadzhikistan or tadzhik or tanzania or tanganyika or thailand or siam or timor leste or east timor or togo or togolese republic or tonga or "trinidad and tobago" or trinidad or tobago or tunisia or turkey or "turkey (republic)" or turkmenistan or turkmen or uganda or ukraine or uruguay or uzbekistan or uzbek or vanuatu or new hebrides or venezuela or vietnam or viet nam or middle east or west bank or gaza or palestine or yemen or yugoslavia or zambia or zimbabwe or northern rhodesia or global south or africa south of the sahara or "sub saharan africa" or subsaharan africa or africa, central or central africa or africa, northern or north africa or northern africa or magreb or maghrib or sahara or africa, southern or southern africa or africa, eastern or east africa or eastern africa or africa, western or west africa or western africa or west indies or indian ocean islands or caribbean region or caribbean islands or caribbean or central america or latin america or "south and central america" or south america or asia, central or central asia or asia, northern or north asia or northern asia or asia, southeastern or southeastern asia or south eastern asia or southeast asia or south east asia or asia, western or western asia or europe, eastern or east europe or eastern europe or developing country or developing countries or developing nation? or developing population? or developing world or less developed countr* or less developed nation? or less developed population? or less developed world or lesser developed countr* or lesser developed nation? or lesser developed population? or lesser developed world or under developed countr* or under developed nation? or under developed population? or under developed world or underdeveloped countr* or underdeveloped nation? or underdeveloped population? or underdeveloped world or middle income countr* or middle income nation? or middle income population? or low income countr* or low income nation? or low income population? or lower income countr* or lower income nation? or lower income population? or underserved countr* or underserved nation? or underserved population? or underserved world or under served countr* or under served nation? or under served population? or under served world or deprived countr* or deprived nation? or deprived population? or deprived world or poor countr* or poor nation? or poor population? or poor world or poorer countr* or poorer nation? or poorer population? or poorer world or developing economy* or less developed economy* or lesser developed economy* or under developed economy* or underdeveloped economy* or middle income economy* or low income economy* or lower income economy* or low gdp or low gnp or low gross domestic or low gross national or lower gdp or lower gnp or lower gross domestic or lower gross national or lmic or lmics or third world or lami countr* or transitional countr* or emerging economies or emerging nation?).ti,ab,sh,kw. | 2134067 |
| 12 | 10 and 11 | 2494 |
| 13 | limit 12 to yr="1990 - 2019" | 2385 |
| 14 | limit 13 to english language | 2238 |

### eFigure 1. PRISMA flow diagram

### eTable 2. Detailed characteristics of included studies for the scoping review

| **Reference** | **Year** | **Country** | **Study type** | **Service setting** | **Population** | **TS as norm or new intervention** | **Study aim** | **TS from** | **TS to** | **Condition** | **Preventive or curative** | **Service shifted** | **Input education** | **Input supervision** | **Input other** | **Outcome/knowledge** | **Outcome/skill** | | **Outcome/patient outcome** | |
| --- | --- | --- | --- | --- | --- | --- | --- | --- | --- | --- | --- | --- | --- | --- | --- | --- | --- | --- | --- | --- |
| **Acute infectious disease and malnutrition** | | | | | | | | | | | | | | | | | | | | |
| (8) | 2017 | Namibia, Kenya, Tanzania, Uganda | Cross-sectional | District hospitals, health centres, dispensaries | Children | Norm | Adherence to IMCI guideline | Doctor or specialist | Non-physician clinician, nurse-midwife, auxiliary staff | Neonatal disorder | Curative | IMCI | Training and re-training in IMCI | 0 | 0 | 0 | Adherence rate higher in physicians and NPCs compared with nurses and auxiliary staff | | 0 | |
| (9) | 2012 | Kenya | Cross-sectional | National referral hospital, secondary and primary level hospital | All population including children | Norm | Describe the case mix, interventions, procedures and patient management of emergency department | Doctor or specialist | Clinical officer | Neonatal disorder | Curative | Acute care investigation, diagnosis and treatment | 3 year training without specific training in emergency medicine | Sometimes clinical officers work alongside medical officer | 0 | 0 | 0 | | 0 | |
| (10) | 1997 | Kenya | Cross-sectional | Maternal and child health clinic | Children under five | Norm | Describe malaria treatment and surveillance in the health facility | Doctor | Clinical officer, community nurse | Malaria | Curative | Malaria screening through blood smear | 0 | 0 | 0 | 0 | 0 | | 0 | |
| (11) | 2012 | Tanzania | Cross-sectional | Paediatric ward and outpatient clinics in regional hospital | Children | Norm | Describe antibiotic prescribe practice for cough and diarrhoea | Doctor or specialist | Clinical officer, assistant medical officer | Neonatal disorder | Curative | Cough and/or diarrhoea treatment | 0 | 0 | 0 | 0 | Inappropriate prescription was more frequent among clinical officers than assistant medical officers and medical officers (48.9% vs. 30.1% and 13.0% respectively) | | 0 | |
| (12) | 2009 | Lesotho | Cohort | One district hospital, 14 clinics | All population including children | Norm | Evaluate outcome of nurse-driven community supported HIV/AIDS treatment program | Doctor | Nurse, nursing assistant | HIV/AIDS | Curative | ART | Quarterly intensive in-service theoretical and practical one week training; Targeted training provided on the basis of weakness identified via evaluations and supervision. | Weekly or bi-weekly visit by doctor or nurse clinician | 0 | 0 | 0 | | Satisfactory outcome with 80% patients still alive and in care by 12 months and 76.5% still in care at 24 months | |
| (13) | 2016 | Nigeria | Cross-sectional | Primary and secondary facilities | Children under five | Norm | Assess knowledge, diagnostics accuracy of health workers | Doctor or specialist | Non-physician clinician including community health officer, nurse officer, nurse midwife, community health extension workers | Neonatal disorder | Curative | IMCI | Training provided | 0 | 0 | Medical officers show significantly higher knowledge of the consultation process, significantly higher diagnostic accuracy, but significantly lower knowledge of the treatment guidelines than non-physician clinicians. | Medical officers asked more physical examination and history taking questions, accurately diagnose cases, prescribed accurate treatment than other cadres | | 0 | |
| (14) | 2014 | Mozambique | Cross-sectional | All types of facility | All population including children | Norm | Task analysis assessing nursing skill mix | Nurse | Medical technician, medical agent | Others | Curative | Nursing care | Pre-service education | 0 | 0 | 0 | 0 | | 0 | |
| (15) | 2014 | Malawi | RCT | Paediatric ward in central hospital | Infant (0-12m) | Norm | Compare WHO algorithm and CD4 count for HIV diagnosis | Specialist | Clinical officer | HIV/AIDS | Curative | HIV/AIDS diagnosis | Half day training in WHO algorithm | 0 | 0 | 0 | Fair agreement level in HIV diagnosis using WHO algorithm, different kappa for other conditions (0.28 for sepsis, 0.62 for oral candida) | | 0 | |
| (16) | 2014 | Kenya | Cross-sectional | Case verification ward in one county | BCG vaccinated infants 0-6 weeks | Norm | Compare interrater agreement and utility of chest radiograph for TB diagnosis (CO was used for initial screening) | Specialist | Doctor, clinical officer | Tuberculosis | Curative | TB screening | Training on reading and identifying TB on radiographs | 0 | 0 | 0 | 0 | | 0 | |
| (17) | 2014 | South Africa | Before-after study | Primary care clinics | All population including children | New | Examine quality of care provided by nurse after the mentorship program | Doctor | Nurse | HIV/AIDS | Curative | ART | HIV training course; 40 hour one-on-one mentorship | 0 | 0 | Increase in nurse graduate's self-assessment | Improvement in quality of nurse managed ART in collection of required blood test, patient HIV staging and nurse adherence assessment and documentation | | 0 | |
| (18) | 2007 | Zambia | Cohort | Primary care clinics | Children | Norm | Report clinical outcomes of children receiving ART | Doctor or specialist | Clinical officer and nurse | HIV/AIDS | Curative | ART | 0 | 0 | 0 | 0 | 0 | | Mortality rate (6.6 per 100) | |
| (19) | 2017 | Myanmar | Cross-sectional | Rural health centre | Newborn | Norm | Assess knowledge of critical danger signs and practices | Midwife | Auxiliary Midwives | Neonatal disorder | Curative | Danger sign | 6 month training and refresher training | Supervision from midwife | 0 | Only 8% of AMWs could identify at least 80% of critical danger signs | 0 | | 0 | |
| (20) | 2013 | Malawi | Cross-sectional | Outpatient district level clinic | Children | Norm | Assess quality of pneumonia care delivered by CO | Doctor or specialist | Clinical officer | Lower respiratory infections | Curative | IMCI - pneumonia care | 3 year education, with or without one year internship | 0 | 0 | 0 | CO correctly diagnosed 30%, administered correct care 25% and failed to hospitalize 40.8% severe pneumonia | | 0 | |
| (21) | 1990 | Lesotho | Cross-sectional | Peripheral health centres | All population including children | Norm | Assess diagnosis and management practice of acute respiratory infection | Doctor or specialist | Nurse clinician, nurse | Lower respiratory infections | Curative | Acute respiratory infection | 18 month course | Supervisory visit | 0 | Nurse-clinicians less likely to prescribe antimicrobial drugs for mild ARI, moderate and severe ALRI; | 0 | | 0 | |
| (22) | 2013 | Mozambique | Before-after study | Clinics | Infant (<=2m) | New | Examine the HIV infection rate before and after option b+ | Clinical officer | Nurse | HIV/AIDS | Curative | ART | 0 | 0 | 0 | 0 | 0 | | Proportion of HIV-exposed infants tested positive reduced from 6% to 4% after option B | |
| (23) | 2019 | Nigeria | Before-after study | Secondary health-care facility | Children and adolescent 0-19y | New | Evaluate effectiveness of task shifting HIV testing services to improve HIV case identification | Lab technician | Nurse | HIV/AIDS | Curative | HIV/AIDS testing | Trained by lab personnel to provide testing | 0 | 0 | 0 | 0 | | Proportion of patient underwent testing increased and newly identified increased. | |
| (24) | 2017 | Kenya | Non-randomized trial | Primary care clinics | All population including children | Norm | Evaluate effect of organizational and educational interventions on adherence to guideline | Doctor or specialist | Clinical officer | Diarrheal disease | Curative | Childhood diarrheal treatment | 3 year pre-service training and one year internship | 0 | A clinical quality assurance system | 0 | Childhood diarrhoea adherence rate for CO 39.9% where control group CO 10.8% | | 0 | |
| (25) | 2019 | South Sudan | Cross-sectional | State hospital | Children < 5 | New | Examine feasibility of training CO to differentiate causes of LRTI | Doctor or specialist | Clinical officer | Lower respiratory infections | Curative | Point-of-care ultrasound for LRTIs | two 6-hour training sessions, including a 1-hour didactic session, a 2-hour bedside teaching session, a 3-hour window to practice using the device. | 0 | 0 | 0 | Experts rated 99.1% acceptable and 86.0% CO interpretation appropriate, kappa was 0.73 for lung consolidation with air bronchograms and 0.81 for viral LRTI | | 0 | |
| (26) | 2013 | Botswana | Cross-sectional | Centre of Excellence | Paediatric patient | Norm | Compare compliance to guideline by nurse and doctor | Doctor | Nurse | HIV/AIDS | Curative | Paediatric HIV treatment including documentation of eight variables | Four weeks of training including didactics and practicum; an additional month of clinical mentorship attached to a licensed physician with paediatric HIV experience. | 0 | 0 | 0 | Nurses and doctors correctly documented 96.0% and 94.9% of the time, respectively. Higher proportion of social history documentation by the nurse though without statistical significance. | | 0 | |
| (27) | 2013 | Sierra Leone | Before-after study | Central government hospital | Neonates | New | Evaluate impact of training on knowledge and skills for essential neonatal care | Doctor or specialist | Nurse, nurse aid, community health officer | Neonatal disorder | Curative | Essential neonatal care | Two day training including interactive lectures, practical demonstrations and small-group facilitated sessions | 0 | Simplified neonatal treatment guideline | Increase in confidence in managing common neonatal problems | 0 | | 0 | |
| (28) | 2013 | Bangladesh | Before-after study | First-level government facilities | Children | New | Examine if quality of care given regular supervision could be sustained | Doctor | Paramedics and female reproductive health worker | Neonatal disorder | Curative | IMCI | 11 day IMCI training | Monthly supervision with checklist | Drug, supplies, recording and reporting system (form), case management guideline | 0 | Both providers performed better in assessing, classifying, correct treatment and prescription following IMCI guideline compared with baseline; improvements in quality of care were sustained over the 2-year period. | | 0 | |
| (29) | 2008 | Tanzania | Cross-sectional | District hospital | Children (2m - 13y) | Norm | Examine inter-observer variation in clinical sign | Doctor or specialist | Clinical officer and nurse | Neonatal disorder | Curative | Paediatric sign examination mostly in IMCI | Video clips that demonstrated the signs in the study; 1-h training session on clinical examination using the same training materials followed an interactive teaching session | 0 | 0 | 0 | Mean (median) Kappa scores for all signs examined were 0.54 (0.57) for RCO–RCO, 0.49 (0.49) for RCO–HCO and 0.50 (0.49) for RCO–HN | | 0 | |
| (30) | 2002 | Tanzania | Cross-sectional | District hospital | Children (4m-6y) | Norm | Examine inter-observer variation in clinical sign | Doctor or specialist | Clinical officer | Neonatal disorder | Curative | Paediatric sign examination mostly in IMCI | Trained to assess clinical signs and complete the surveillance forms in a standardized way, | Daily supervision before study commenced | 0 | 0 | At least fair agreement in most clinical signs between COs | | 0 | |
| (31) | 2015 | Mozambique | Cohort | Secondary health care facilities | Children | Norm | Describe long-term treatment outcomes of paediatric HIV cohort | Doctor or specialist | Nurse | HIV/AIDS | Curative | Followup of HIV/AIDS | 0 | Close program supervision was conducted by a referent physician who was also in charge of ART initiation and diagnosis, and the treatment of complicated cases. | 0 | 0 | 0 | | 6% of children who initiated ART died, 15% loss to follow-up. | |
| (32) | 2007 | Malawi | Case study/review | NA | All population including children | Norm | Describe achievement and challenge of ART scale-up | Doctor or specialist | Clinical officer, nurse, medical assistant | HIV/AIDS | Curative | ART | 5-day training module and a certification of competence linked to a formal exam- nation, and successful participants then undertook a 2-week practical clinical attachment at one of the experienced ART sites | 0 | 0 | 0 | 0 | | 74% alive and on ART at the initial ART registration site, 10% dead, though not all children | |
| (33) | 2012 | Malawi | Cross-sectional | the Martin Preuss Centre (MPC) and the Lighthouse clinic at Kamuzu Central Hospital | Children <15y | Norm | Compare nurse and clinical officer practice in ART refill, adherence, etc. | Doctor or specialist | Clinical officer and nurse | HIV/AIDS | Curative | ART | Nurses underwent a two-hour refresher training on the paediatric dosing charts, adherence and appointment calculation and entered a three-day pilot phase with intense supervision by the study coordinator to clarify questions and to ensure that all operations followed the study protocol | 0 | Formulas and dosage charts | 0 | Agreement rate in response to questions on adherence, dose, formulation and next appointment was high | | 0 | |
| (34) | 2017 | Bangladesh | Non-comparative evaluation | First level referral sub-district hospitals | Children < 5 | New | Quality improvement in managing neonatal and early childhood syndromic sepsis | Doctor or specialist | Paramedics and nurse | Neonatal disorder | Curative | Assess, identify and admit of sepsis | One day training and refresher (quarterly in year one and six monthly in year two) to reinforce education and promote knowledge retention | 0 | Standard operating procedure, flow chart | 0 | 0 | | Significant improvement in the rate of establishing vascular access and choice of recommended first line parenteral antibiotic over time, increase in proportion of patients discharged with medical advice. | |
| (35) | 2012 | Malawi | Cross-sectional | NA | Children | Norm | Compare ART prescription between nurse and clinician | Doctor | Nurse | HIV/AIDS | Curative | ART | 0 | 0 | 0 | 0 | Good agreement rate and mean differences between nurses and clinician were -0.005 and -0.009 for morning and evening dosage | | 0 | |
| (36) | 2019 | Cameroon | Cross-sectional | Health facilities | Children | Norm | Describe knowledge, attitude and practices of health workers regarding paediatric HIV | Doctor or specialist | Nurse, nurse's aid, assistant lab technician | HIV/AIDS | Curative | HIV paediatric care including ART | 0 | 0 | 0 | Knowledge on HIV transmission, treatment, PMTCT was acceptable though only 5.2% of staff knew that HIV-infected infants aged less than 2 years were an indication initiation of ART | Viral load exam as a follow-up biomarker was not requested by 40.7% of the health workers | | 0 | |
| (37) | 2017 | Cote d'Ivoire | Non-randomized trial | Primary and secondary facility | All population including children | New | Evaluate feasibility of nurse-led HIV care | Doctor or specialist | Nurse | HIV/AIDS | Curative | ART | 1 month training on HIV management and ART initiation | supervising physician visiting the facility on a weekly or twice- a-week basis to provide mentorship and review cases. | 0 | 0 | 0 | | Nursing with visiting physician has 5.3% mortality vs. nurse with onsite physician 3.6% mortality, retained rate 89.9% vs 82.7% | |
| (38) | 2015 | South Africa | Before-after study | Primary care facilities | Children | Norm | Evaluate the effect of automated IMCI guideline on training outcomes | Doctor or specialist | Nurse | Neonatal disorder | Curative | IMCI | Three four hour workshops over three non-consecutive days on IMCI | 0 | Automated version of IMCI guideline | Knowledge test score improved for both control and intervention group | Nurses in the automated group performed significantly better in use of the IMCI guideline (p < 0.05): checking immunisations (68% vs. 93%), making a complete assessment (62% vs 100%), prescribing correct medication (50% vs 85%) and correct dose (42% vs 85%). | | 0 | |
| (39) | 2014 | Uganda | Non-comparative evaluation | Laroli Lwanga Hospital | All population including children | New | Describe patient outcome treated by student ECPs | Doctor or specialist | Emergency care practitioner | Neonatal disorder, malaria | Curative | Acute care including malaria, trauma, pneumonia | 2 year education using semi-weekly didactic, simulation, etc. | Supervised by emergency physician | 0 | 0 | 0 | | Case fatality rate 2.3% and 2.6% for malaria and pneumonia | |
| (40) | 2008 | Rwanda | Non-comparative evaluation | Health centres | Children | Norm | Describe experience of nurse-based care for ART for children | Doctor or specialist | Nurse | HIV/AIDS | Curative | ART | theoretical and bed-side training in comprehensive HIV care in general, and in paediatric aspects | Mentoring and supervision by physician | 0 | 0 | 0 | | Mortality 2.6% during follow-up, satisfactory viral suppression in 86.8%, safety (toxicity for ART) 8.3% | |
| (41) | 2009 | South Africa | Cohort | Primary healthcare clinics | Children | Norm | Describe clinical outcomes of decentralized nurse-counsellor-led ART program | Doctor or specialist | Nurse and lay staff | HIV/AIDS | Curative | ART | Standardised training on issues including HIV counsel- ling and testing, HIV treatment and adherence, and drug side- effect | 0 | 0 | 0 | | Mortality (6.7%), including 53.1% within 90 days of treatment initiation. CD4% and other parameters. | |  |
| (42) | 2013 | DR Congo | Cohort | Primary healthcare clinics | children | Norm | Describe clinical outcomes of tuberculosis and HIV for children at primary health care level | Doctor or specialist | Nurse | HIV/AIDS; Tuberculosis | Curative | HIV/AIDS and TB treatment | Trained | 0 | Standard algorithms | 0 | 0 | | Mortality (6.5%) during anti-tuberculosis treatment, 74.2% initiated ART; CD4 count and BMI increased for children | |
| (43) | 2009 | Zambia | Case study/review | NA | All population including children | New | Describe experience of using task shifting to scale up HIV/AIDS treatment service | Doctors and clinical officers | Clinical officers and nurses | HIV/AIDS | Curative | Initial consultation, lab test, ART, triage, etc | Didactic sessions targeting specialized skill sets followed by an intensive period of practical mentorship, where providers are paired with trainers before working independently | 0 | Targeted chart review, monthly site report, feedback and training, exchange programme | 0 | 0 | | 0 | |
| (44) | 2018 | Malawi | Interview | Primary care clinics | Children | Norm | Understand how IMCI and ETAT was integrated in a primary care facility | Doctor or specialist | Medical assistance | Neonatal disorder | Curative | IMCI and ETAT | IMCI training | 0 | 0 | 0 | 0 | | 0 | |
| (45) | 2018 | South Sudan | Case study/review | Primary care center | Newborn | Norm | Describe factors influencing newborn care implementation | Doctor or specialist | Nurse, clinical officer, midwife | Neonatal disorder | Curative | Danger signs during pregnancy, fetal monitoring using the partograph, neonatal resuscitation, immediate newborn care, breastfeeding support, recognition of newborn danger signs, feeding and kangaroo mother care (KMC) for small babies, management of possible severe infections, and postnatal counselling before patient discharge. | Training session including danger signs during pregnancy, fetal monitoring using the partograph, neonatal resuscitation, immediate newborn care, breastfeeding support, recognition of newborn danger signs, feeding and kangaroo mother care (KMC) for small babies, management of possible severe infections, and postnatal counselling before patient discharge | Supervision with checklist: A checklist was designed for supervisors to observe practices learned in the clinical training during a delivery or home visit. Based on the observation, the supervisor provided feedback to the health worker and recorded areas in need of further attention | Medical commodities i.e. newborn medical supply kits | 0 | 0 | | 0 | |
| (46) | 2017 | Kenya | Interview | Public clinics | Children | Norm | Assess knowledge and practices of clinicians when assessing children with fever | Doctor or specialist | Clinical officer, nurse | Others | Curative | Fever including malaria and non-malaria illness | 0 | 0 | 0 | 0 | 0 | | 0 | |
| (47) | 2019 | Burkina Faso | Interview | Primary health care facilities | Children | Norm | Understand clinicians' view on eIMCI tools | Doctor or specialist | Health officer, nurse, midwife, birth attendant | Neonatal disorder | Curative | IMCI | 0 | 0 | Electronic tool guideline including electronic algorithm based on IMCI | 0 | 0 | | 0 | |
| (48) | 2015 | Ethiopia | Before-after study | District hospital | Newborn | New | Describe training outcome of two day training on newborn resuscitation and care | Doctor or specialist | Nurse, clinical officer, midwife | Neonatal disorder | Curative | Newborn resuscitation and care | Two day training using country and regional guideline, lecture, group work, skill teaching scenario | 0 | 0 | Written test score improved from 53.2% to 66.4%; practical test score post course 5.5 out of 6 | 0 | | 0 | |
| Acute infectious disease and malnutrition + Surgery, injury and intensive care | | | | | | | | | | | | | | | | | | | | |
| (49) | 2015 | Uganda | Non-comparative evaluation | District hospital | All population including children | New | Describe mortality rate of patients treated by task shifted midlevel provider | Doctor or specialist | Emergency care practitioner (nurse) | Other (Emergency care) | Curative | Acute illness and injury including 9 diagnosis (most common malaria, pneumonia, trauma, malnutrition) | Initially paired with a physician certified in emergency medicine for nine months, continued teaching by rotating volunteer physicians | 0 | 0 | 0 | 0 | | Under-five case fatality rate were 1.9% for malaria, 4.1% for pneumonia, 1.6% for trauma and 6.8% for malnutrition | |
| (50) | 2019 | Ghana | Before-after study | NA | Children | New | Evaluate training outcome of adding trauma alongside ETAT course | Doctor or specialist | Physician assistant, nurse, midwife | Neonatal disorder | Curative | ETAT and trauma | ETAT+ course and one module of trauma teaching | 0 | 0 | Confidence increased for injury management after training (3.75 pre and 4.19 8 weeks post), knowledge also increased for injury management (3.82 pre and 4.25 8 weeks post) | 0 | | 0 | |
| **Surgery, injury and intensive care** | | | | | | | | | | | | | | | | | | | | |
| (51) | 2015 | South Africa | Case study/review | Regional hospitals, rural clinics, urban clinics | All population including children | Norm | Describe availability of primary and secondary burn service | Doctor or specialist | Doctor, nurse | Fire, heat, and hot substances | Curative | Burn service | 0 | 0 | Referral to higher level facility | 0 | 0 | | 0 | |
| (52) | 2014 | Malawi | Non-randomized trial | Central hospital | Children and adolescent (<=17y) | New | Compare paediatric surgical cases between physician and clinical officer | Specialist | Clinical officer | Fire, heat, and hot substances; neonatal disorder | Curative | Burn surgery, neurosurgery, ENT procedures, congenital, VP shunt placement, foreign body removal, general surgery cases such as laparotomy, appendectomy, and incarcerated hernia repair, as well as minor procedures including biopsy and incision and drainage | 3 year education and one year rotation clinical internship | Oversight and supervision | 0 | 0 | 0 | | Similar reoperation rate (7.1% for MD, 17% for CO), complication rate (4.5% vs. 4.0%), mortality rate (2.5% vs. 2.1%), and length of stay (10 vs. 24d) considering case mix. Burn cases usually are managed by Cos therefore have longer length of stay for excision and grafting. | |
| (53) | 2019 | Kenya | Non-comparative evaluation | Level 4 and 6 hospitals | Neonate and paediatric patients | New | Examine if training of trainers can increase skills and knowledge of health care providers | Specialist | Doctor, nurse, clinical officer | Neonatal disorder | Curative | CPAP | 2 day training session | 0 | 0 | Knowledge score 91% and 90% for 1st and 2nd generation trained providers | Skill score 90% and 89% for 1st and 2nd generation trained providers | | Total mortality rate 24%, 95% no adverse event | |
| (54) | 2011 | Malawi | Non-randomized trial | Central hospital | All population including children | New | Examine outcome of major general surgery performed by clinical officer | Specialist | Clinical officer | Neonatal disorder | Curative | VP shunting | 3 year pre-service training and one year internship | Study compared with supervision and without supervision | 0 | 0 | 0 | | Postoperative mortality rates (6.6% vs 5.9%), wound infection rates (3.3% vs 3.9%), rates of early shunt revision (0 vs. 3.9%) and shunt removal in CO only and surgeron present group. Length of stay shorter in surgeon present group though could due to patients discahrged on weekends without regular ward rounds | |
| (55) | 2016 | Uganda | Before-after | Emergency department at Karoli Lwanga “Nyakibale” Hospital | Children < 5 | New | Compare in-hospital mortality rate of children under five receiving emergency care by non-physician clinician (a new cadre) alone or supervised by physician | Specialist | Emergency care practitioner (nurse, new cadre) | Other (Emergency care) | Curative | Emergency medicine for patients (including but not limited to malnourishment, hypoxias and severe anaemic, etc.) | Initially paired with a physician certified in emergency medicine for nine months, continued teaching by rotating volunteer physicians | initially supervised by a physician and subsequent care was independent | 0 | 0 | 0 | | 3 day in hospital mortality rate 5.04% for unsupervised, 2.90% for supervised. For majority of patients that not severely ill mortality rate showed no difference (2.17% vs. 3.09%) | |
| (56) | 2005 | Malawi | Non-comparative evaluation | NA | Children | New | Assess outcome of CO performed idiopathic clubfoot deformity | Doctor or specialist | Clinical officer | Congenital birth defects | Curative | Clubfoot deformity | 3 day residential course on how to recognize and manage idiopathic clubfoot, and practical workshop that involved practicing casting techniques on dummy feet reinforced the theoretical lectures on the Ponseti technique | One-on-one teaching and supervision offered | 0 | 0 | 0 | | 98 of 100 clubfeet in our study were corrected to plantigrade or better by OCOs, 2% were referred for corrective surgery having failed treatment by conservative treatment | |
| (57) | 2017 | Malawi | Non-randomized trial | Central hospital | All population including children | New | Examine outcome of orthopaedic clinical officer vs. surgeon present in major amputations, open reductions and plating | Specialist | Clinical officer | Other (Surgery) | Curative | Major amputation, open reduction and internal fixation with plates | Diploma in clinical orthopaedics including a structured training of 18 months following a minimum of four years working experience as a Medical Assistant | Previous working experience under close supervision for years; study compared with supervision and without supervision | 0 | 0 | 0 | | Peri-operative outcome between CO alone and surgeon present group: death 15.6% vs 12.9%, blood transfusion 32.5% vs. 41.9%, infection 16.9% vs. 19.4%, re-operation 15.6% vs. 19.4%, length of stay 18d vs 20d | |
| (58) | 2014 | Malawi | Cost effectiveness | District hospital | All population including children | Norm | Assess the cost-effectiveness of orthopaedic clinical officer programme | Doctor or specialist | Clinical officer | Other (Surgery) | Curative | Amputation, fracture, etc. | 0 | 0 | 0 | 0 | 0 | | Total cost-effectiveness of providing orthopaedic care through the OCO training programme was US$92.06 per DALY averted | |
| (59) | 2013 | Uganda | Cross-sectional | District hospital | All population including children | Norm | Quantify the effect of geography on patient outcomes | Doctor or specialist | Non-physician clinician | Other (Emergency care) | Curative | Emergency medicine | 0 | 0 | 0 | 0 | 0 | | 0 | |
| (60) | 2013 | Zambia | Non-randomized trial | One University Teaching Hospital and two primary health care clinics | Neonate (aged 0-28d, Gestational age >37w, weight 2500-5000g) | New | Compare the neonatal circumcision outcome of three different devices | Doctor or specialist | Doctor, nurse midwife, clinical officer, nurse | HIV/AIDS | Preventive | Neonatal male circumcision | Training package consist of didactic lectures, practice on models of neonatal genitalia and clinical practice | 0 | 0 | 0 | Nurses took longer to train than other providers though not statistically significant | | Total adverse event rate 4.9% though including performed by physicians | |
| (61) | 2017 | Uganda | Non-randomized trial | Government health centres | Infant (1-28d) | New | Examine knowledge and competence in early infant male circumcision after training | Doctor or specialist | Clinical officer, nurse-midwife | HIV/AIDS | Preventive | Early infant male circumcision | 5 day didactic training, and hands-on surgical training on 15 cases | 0 | 0 | Both (CO and RNMW)' knowledge score increased. CO's knowledge score higher than RNMW after training (100% vs. 60%) | Competency score increased for both group though modest. | | Pain score similar in two groups, adverse event rate 3.5% | |
| (62) | 2014 | Kenya | Non-randomized trial | Hospital, health centre and dispensary | All population including adolescent | New | Evaluate safety of male circumcision performed by non-physician clinician | Doctor or specialist | Clinical officer and nurse | HIV/AIDS | Preventive | Male circumcision | Training program on WHO/UNAIDS "Manual for Male Circumcision Under Local Anaesthesia" | 0 | 0 | 0 | 0 | | Adverse event rate (2.1% for nurses and 1.9% for clinical officers) and client satisfaction over 99% | |
| (63) | 2012 | Kenya | Non-comparative evaluation | Government health facilities | Infant < 2m | New | Evaluate safety of infant male circumcision | Doctor or specialist | Clinical officer and nurse | HIV/AIDS | Preventive | Early infant male circumcision | 0 | 0 | 0 | 0 | 0 | | Adverse event rate (2.7%) and patient satisfaction rate 96% | |
| (64) | 2012 | Kenya | Non-comparative evaluation | NA | All population including children | New | Examine service trend in voluntary male circumcision | Doctor or specialist | Clinical officer and nurse | HIV/AIDS | Preventive | Male circumcision | 0 | 0 | 0 | 0 | 0 | | Adverse event rate 1.4% for CO and nurse respectively and 0% for MO | |
| (65) | 2019 | Kenya | RCT | NA | All population including adolescent | Norm | Compare safety of MC device vs. topical anaesthesia in male circumcision | Doctor or specialist | Non-physician clinician | HIV/AIDS | Preventive | Male circumcision | 0 | 0 | 0 | 0 | 0 | | Mean pain score similar in two treatment groups, no adverse event, mean operate time and rate of complete wound healing similar across two groups | |
| (66) | 2016 | Uganda | RCT | Health centre | Infant (1-28d) | New | Assess safety and acceptability of early infant circumcision by CO and nurse midwives | Doctor or specialist | Clinical officer, nurse-midwife | HIV/AIDS | Preventive | Early infant male circumcision | Trained though no detail of training | 0 | 0 | 0 | 0 | | Adverse event rate low (2.4% and 1.6% for CO and NW) and maternal satisfaction high (99.6% and 100% respectively) | |
| Chronic conditions | | | | | | | | | | | | | | | | | | | | |
| (67) | 2018 | Uganda | Before-after study | Lower level health centres | Children | New | Assess impact of training program on knowledge and practice for RHD prevention | Doctor or specialist | Clinical officer, nurse, nurse assistant, midwife | Rheumatic heart disease | Curative | RHD prevention and monthly benzathine penicillin G administration | 3 month RHD education training program | 0 | 0 | Knowledge score regarding recognition and prevention of acute rheumatic fever improved (from 10% and 7.5% for recognition and prevention of ARF to 72.5% and 50%) | 0 | | BPG adherence level remained similar and without any adverse event following BPG decentralization (95.8% vs 94.5) | |
| (68) | 2015 | Malawi | Cross-sectional | NA | Children | New | Examine agreement rate between paediatric cardiologist and clinical officer for RHD screening | Specialist | Clinical officer | Rheumatic heart disease | Preventive | RHD screening using ECG | 3 half day didactic and computer module based training and 2 days clinical attachment | 0 | 0 | 0 | Kappa between specialist and CO was 0.72; overall sensitivity 0.92, specificity 0.80 | | 0 | |
| (69) | 2019 | Malawi | Before-after study | Central hospital | All population including children | New | Examine the outcome of an education program on RHD | Doctor or specialist | Doctor, nurses, clinical officer | Rheumatic heart disease | Curative | RHD treatment | 3 half-day workshop | 0 | 0 | Improvement in knowledge score from 43.8% to 78.5% and more comfortable prescribing or injecting benzathine penicillin | 0 | | 0 | |
| (70) | 2018 | Rwanda | Cross-sectional | District clinic | All population including children | New | Describe validity (diagnosis accuracy) of nurse-diagnosis of heart failure and patient outcomes | Specialist | Nurse | Rheumatic heart disease | Curative | Echocardiography diagnosis and heart failure treatment based on algorithms | 0 | 0 | 0 | 0 | Nurse-performed echocardiography had sensitivity and specificity of 81% and 91% for other RHD; | | 0 | |
| (71) | 2012 | Brazil | Case study/review | Referral hospital | Children and adolescent | New | Describe Brazil experience of clinical nurse specialist | Doctor or specialist | Nurse specialist | Brain and nervous system cancer | Curative | CNS tumour care, education, research and management | 0 | 0 | 0 | 0 | 0 | | 0 | |
| (72) | 2017 | Malawi | Cross-sectional | Schools | Children (5-16y) | New | Compare clinical officer and paediatric cardiologist agreement rate for RHD diagnosis | Specialist | Clinical officer | Rheumatic heart disease | Curative | RHD screening | Three half-days of didactic lectures and computer-based training module, after completion of classroom training, each clinical officer spent 2 hours learning basic practical image acquisition skills on volunteer patients under supervision. | 0 | 0 | 0 | mean kappa statistic comparing clinical officer referrals with the paediatric cardiologist was 0.72; sensitivity 0.91, specificity 0.65 | | 0 | |
| (73) | 2016 | Brazil | Cross-sectional | Schools | Children | New | Evaluate performance of non-experts in school-based echo-screening programme for RHD | Doctor or specialist | Nurse and health technician | Rheumatic heart disease | Curative | RHD screening | Standardized, computer-based training | 0 | 0 | 0 | Sensitivity and specificity 85% and 87% when using MR>=1.5 cm/any AI, 64% and 96% when using MR>=2.0 cm/any AI | | 0 | |
| (74) | 2015 | Fiji | Cross-sectional | NA | All population including children | New | Evaluate knowledge of training on echocardiography for RHD screening | Doctor or specialist | Nurse | Rheumatic heart disease | Curative | RHD screening | Classroom training for one week (relevant cardiac anatomy and physiology, as well as basic knowledge about RHD and the rationale for screening), practical session (essentials of using the machines, and practice with volunteer children, including some with known RHD), interactive tutorial | 0 | 0 | Knowledge score increased from 8.1 to 14.9 (out of 15) after training, 98% nurses of adequate quality for diagnosis | 0 | | 0 | |
| (75) | 2013 | Fiji | Cross-sectional | NA | Children | New | To determine whether nurses could follow an algorithm to identify cases of RHD | Doctor or specialist | Nurse | Rheumatic heart disease | Curative | RHD screening | A week-long rheumatic heart disease echo cardiography training workshop, 2 weeks of echocardiography screening in two primary schools supervised by experienced doctors and echo technicians. | 0 | Eleven-step basic algorithm for nurse-led RHD screening echocardiography. | 0 | sensitivity of 100% and 83%, and a specificity of 67.4% and 79%, respectively for the two nurses | | 0 | |
| (76) | 2016 | Uganda | Cross-sectional | Schools | Children | New | To examine nurse performance and interpretation of handheld echocardiography for RHD screening | Doctor or specialist | Nurse | Rheumatic heart disease | Preventive | RHD screening | 4-h of physician-directed teaching using a combination of computer-based training modules, didactics and case studies; 2-day hands on session with patients. | 0 | 0 | 0 | sensitivity of 74.4% (58.8% to 86.5%) and a specificity of 78.8% (76.0% to 81.4%) for any RHD (borderline and definite). | | 0 | |
| (77) | 2017 | Tanzania | Case study/review | National referral hospital | All population including children | New | Describe the development of haematology nurse education to strengthen nurse capacity | Doctor or specialist | Nurse | Hemoglobinopathies and haemolytic anaemias | Preventive and curative | Haematology related service | 2 week collaborative education program: (1) basic haematology and haemato-oncology group lectures; (2) individual instruction on self-identified needs; (3) direct observation of patient care in paediatric and adult inpatient wards and haematology outpatient clinics; and (4) group lectures to graduate and postgraduate nursing students. | 0 | 0 | 0 | 0 | | 0 | |
| (78) | 2016 | Kenya | Non-comparative evaluation | Primary healthcare facility | All population including children | New | Evaluate adherence to protocol for five "stable" NCDs when shifted from clinical officer to nurse | Clinical officer | Nurse | Epilepsy; Hemoglobinopathies and haemolytic anaemias | Curative | Epilepsy management, sickle cell | One week with didactic and clinical case scenario | Supervising clinical officer and field mentorship | Structured clinical support tool | 0 | Adherence to protocol for epilepsy: patient consultation (82%), weight checked (55%) | | 0 | |
| (79) | 2016 | South Africa | RCT | Schools | Adolescent (13-18y) | New | Compare two different treatment for PTSD both delivered by nurse | Specialist | Nurse | Anxiety disorder | Curative | Prolonged exposure or supportive counselling for PTSD | 1-year advanced psychiatry diploma, 4-day workshop, 16 hour practical training | Group supervision every week | 0 | 0 | 0 | | Both treatment arms (both performed by nurse) had significant decrease in PTSD and depression post treatment | |
| (80) | 2013 | Uganda | Case study/review | Satellite clinic in the local village | Children | New | Describe case of setting up satellite clinic to prove epilepsy treatment | Specialist | Clinical officer | Epilepsy | Curative | Epilepsy treatment | Extra training in epilepsy | 0 | 0 | 0 | 0 | | 70% attending for follow-up in satellite clinics, whereas in hospital the majority did not attending follow-up | |
| (81) | 2017 | Uganda | Before-after study | Primary heath care centres | Child and adolescent | New | Describe learning outcomes after an in-service training | Doctor or specialist | Clinical officer, nurse, midwife | Mental disorder, substance use disorder, epilepsy | Curative | Identification of mental, neurological or substance use disorder | 5 day residential training including classroom and practicum | 0 | 0 | Improvement in mean test score for mental health knowledge, clinical officers had higher mean score | 0 | | 0 | |
| (82) | 2019 | Zambia | Before-after study | Peri-urban first-level health centre | Children | New | Examine changes in knowledge for epilepsy after training | Doctor or specialist | Clinical officer | Epilepsy | Curative | Epilepsy diagnosis and management | 3-week six training model and open case discussion | 0 | 0 | Knowledge of epilepsy: medication management, recognition of focal seizure, history taking, medication titration increased; knowledge regarding provoked seizures, use of diagnostic studies and general aetiologies of epilepsy remained limited | 0 | | 0 | |
| (83) | 2018 | South Africa | RCT | Schools | Adolescent (13-18) | New | Evaluate effectiveness of task shifted intervention for PTSD, both group performed by nurse | Specialist | Nurse | Anxiety disorder | Curative | Prolonged exposure or supportive counselling for PTSD | 1-year advanced psychiatry diploma, 4-day workshop, 16 jour practical training | Group supervision every week | 0 | 0 | 0 | | Both treatment arms (both performed by nurse) improved patient PTSD (interviewer-rated from 35.32 to 9.29 at 6 month), depression (from 31.4 to 10.12 at 6 month), global functioning (from 52.01 to 67.26 at 6 month) | |
| (84) | 2008 | Cameroon | Case study/review | Clinic | All population including children | New | To examine nurse-led care for epilepsy at primary level in rural health district | Doctor or specialist | Nurse | Epilepsy | Curative | Epilepsy treatment (Phenobarbital and phenytoin) | Trained | Physician available as needed for advice | Dosage chart and protocol | 0 | 0 | | Total mortality rate 2.7% and reduced seizure during follow-up period | |
| (85) | 2008 | Cameroon | Non-randomized trial | Clinic | All population including children | New | To examine nurse-led care for asthma at primary level in rural health district | Doctor or specialist | Nurse | Asthma | Curative | Asthma diagnosis and treatment | 4-day intensive training with demonstration at the beginning of the program and another refresher course was organized a year later | Physician available as needed for advice | Clinical management algorithm | 0 | 0 | | Median follow-up 2 visits, 39.1% re-hospitalization rate, no death in child and adolescent group | |
| (86) | 2014 | Ethiopia | Case study/review | Specialized hospital | Child and adolescent | New | Describe the development and implementation of child psychiatry program for non-physician clinicians | Doctor or specialist | Non-physician clinician | Mental disorder | Curative | Child psychiatry | Two week course, main emphasis on how to: assess the emotions, behaviour and functioning of children; develop management plans for specific mental health conditions, and engage and work with patients, families and other providers based on the WHO mhGAP-IG guidelines and four week internship | 0 | 0 | 0 | Participants of the course have been seeing child patients since completing the child course, and express confidence in caring for child patients. | | 0 | |
| (87) | 2019 | Uganda | Case study/review | Primary health care facilities | Child and adolescent | Norm | Assess adherence to standard for nodding syndrome and identify gaps in care | Doctor or specialist | Clinical officer, nurse and other PHC provider | Epilepsy | Curative | Epilepsy treatment | 0 | 0 | 0 | 0 | 0 | | 0 | |
| (88) | 2017 | South Africa | Interview | Schools | Adolescent (13-18) | New | Understand impediments and catalysts for task shifting intervention | Specialist | Nurse | Anxiety disorder | Curative | Prolonged exposure or supportive counselling for PTSD | 1-year advanced psychiatry diploma, 4-day workshop, 16 jour practical training | Group supervision every week | 0 | 0 | 0 | | 0 | |
| (89) | 2018 | South Africa | Interview | Schools | Adolescent (13-18y) | New | Experience of nurse provider and adolescents of task-shifting trial participation | Specialist | Nurse | Anxiety disorder | Curative | Prolonged exposure or supportive counselling for PTSD | 1-year advanced psychiatry diploma, 4-day workshop, 16 hour practical training | Group supervision every week | 0 | 0 | 0 | | 0 | |
| (90) | 2014 | Tanzania | Before-after study, interview | Public reproductive and child health clinics | Children | New | Evaluate implementation of 10 key activities for healthy eyes delivered by primary level staff | Doctor or specialist | Clinical officer, nurses, students | Blindness and vision impairment | Preventive and curative | Eye care prevention/prophylaxis, control of ocular conditions | Training in 10 key activities and educational materials provided | 0 | Referral and torch for examination | Trained staff could name more eye conditions of childhood than untrained staff | Diagnostics skills (correctly identified conjunctivitis and cataract) better in trained staff than control | | Management (referral) of cataract and trauma better in trained staff than control | |

##

## Reference

1. Arksey H, O’Malley L. Scoping studies: towards a methodological framework. Int J Soc Res Methodol. 2005 Feb 1;8(1):19–32.

2. WHO | Task shifting: global recommendations and guidelines [Internet]. WHO. World Health Organization; [cited 2020 May 15]. Available from: https://www.who.int/workforcealliance/knowledge/resources/taskshifting_guidelines/en/

3. WHO | Definition of key terms [Internet]. WHO. World Health Organization; [cited 2020 May 20]. Available from: https://www.who.int/hiv/pub/guidelines/arv2013/intro/keyterms/en/

4. LMIC Filters [Internet]. [cited 2021 Feb 9]. Available from: /lmic-filters

5. Wallace BC, Small K, Brodley CE, Lau J, Trikalinos TA. Deploying an interactive machine learning system in an evidence-based practice center: abstrackr. In: Proceedings of the 2nd ACM SIGHIT International Health Informatics Symposium [Internet]. New York, NY, USA: Association for Computing Machinery; 2012 [cited 2020 Sep 9]. p. 819–24. (IHI ’12). Available from: https://doi.org/10.1145/2110363.2110464

6. Cicchetti DV, Feinstein AR. High agreement but low kappa: II. Resolving the paradoxes. J Clin Epidemiol. 1990 Jan 1;43(6):551–8.

7. Gwet KL. Handbook of Inter-Rater Reliability, 4th Edition: The Definitive Guide to Measuring The Extent of Agreement Among Raters. Advanced Analytics, LLC; 2014. 429 p.

8. Krüger C, Heinzel-Gutenbrunner M, Ali M. Adherence to the integrated management of childhood illness guidelines in Namibia, Kenya, Tanzania and Uganda: evidence from the national service provision assessment surveys. BMC Health Serv Res. 2017 Dec;17(1):822.

9. Wachira BW, Wallis LA, Geduld H. An analysis of the clinical practice of emergency medicine in public emergency departments in Kenya. Emerg Med J. 2012 Jun;29(6):473–6.

10. Some ES, Koech DK, Ochogo JO, Ocholla F, Mumbi F. An evaluation of surveillance of malaria at primary health care level in Kenya. East Afr Med J. 1997 Sep;74(9):573–5.

11. Gwimile JJ, Shekalaghe SA, Kapanda GN, Kisanga ER. Antibiotic prescribing practice in management of cough and/or diarrhoea in Moshi Municipality, Northern Tanzania: cross-sectional descriptive study. :8.

12. Cohen R, Lynch S, Bygrave H, Eggers E, Vlahakis N, Hilderbrand K, et al. Antiretroviral treatment outcomes from a nurse-driven, community-supported HIV/AIDS treatment programme in rural Lesotho: observational cohort assessment at two years. J Int AIDS Soc. 2009 Feb;12(1):23–23.

13. Villar Uribe M. As Good As Doctors?: Task-Shifting Primary Care to Non-Physician Clinicians in Nigeria [Internet] [Thesis]. Johns Hopkins University; 2016 [cited 2021 Mar 7]. Available from: https://dspace-prod.mse.jhu.edu/handle/1774.2/39670

14. Dgedge M, Mendoza A, Necochea E, Bossemeyer D, Rajabo M, Fullerton J. Assessment of the nursing skill mix in Mozambique using a task analysis methodology. Hum Resour Health. 2014 Dec;12(1):5.

15. Maliwichi M, Rosenberg NE, Macfie R, Olson D, Hoffman I, van der Horst CM, et al. CD4 count outperforms World Health Organization clinical algorithm for point-of-care HIV diagnosis among hospitalised HIV-exposed Malawian infants. Trop Med Int Health. 2014 Aug;19(8):978–87.

16. Kaguthi G, Nduba V, Nyokabi J, Onchiri F, Gie R, Borgdorff M. Chest Radiographs for Pediatric TB Diagnosis: Interrater Agreement and Utility. Interdiscip Perspect Infect Dis. 2014;2014:1–8.

17. Green A, de Azevedo V, Patten G, Davies M-A, Ibeto M, Cox V. Clinical Mentorship of Nurse Initiated Antiretroviral Therapy in Khayelitsha, South Africa: A Quality of Care Assessment. Sued O, editor. PLoS ONE. 2014 Jun 2;9(6):e98389.

18. Bolton-Moore C, Mubiana-Mbewe M, Cantrell RA, Chintu N, Stringer EM, Chi BH, et al. Clinical Outcomes and CD4 Cell Response in Children Receiving Antiretroviral Therapy at Primary Health Care Facilities in Zambia. JAMA. 2007 Oct 24;298(16):1888.

19. Than KK, Morgan A, Pham MD, Beeson JG, Luchters S. Determinants of knowledge of critical danger signs, safe childbirth and immediate newborn care practices among auxiliary midwives: a cross sectional survey in Myanmar. BMJ Open. 2017 Jun;7(6):e017180.

20. Bjornstad E, Preidis GA, Lufesi N, Olson D, Kamthunzi P, Hosseinipour MC, et al. Determining the quality of IMCI pneumonia care in Malawian children. Paediatr Int Child Health. 2014 Feb;34(1):29–36.

21. Redd S, Moteetee M, Waldman R. Diagnosis and management of acute respiratory infections in Lesotho. Health Policy Plan. 1990;5(3):255–60.

22. Sebastian T, Brusamento S, Ahoua L, Aly D, Arpadi S, Teasdale CA, et al. Early HIV infection rate trends in exposed infants pre & post Option B+ in Mozambique. :1.

23. Ofem O, Jasper TL, Torbunde N, Yashimankut M, Sadiq H, Ejekam R, et al. Effect of task-shifting HIV testing from laboratory personnel to nurses on paediatric and adolescent HIV testing rate and yield in rural Nigeria: a prospective before-and-after study. Lancet Glob Health. 2019 Mar;7:S23.

24. Egger JR, Stankevitz K, Korom R, Angwenyi P, Sullivan B, Wang J, et al. Evaluating the effects of organizational and educational interventions on adherence to clinical practice guidelines in a low-resource primary-care setting in Kenya. Health Policy Plan. 2017 Jul;32(6):761–8.

25. Nadimpalli A, Tsung JW, Sanchez R, Shah S, Zelikova E, Umphrey L, et al. Feasibility of Training Clinical Officers in Point-of-Care Ultrasound for Pediatric Respiratory Diseases in Aweil, South Sudan. Am J Trop Med Hyg. 2019 Sep 4;101(3):689–95.

26. Monyatsi G, Mullan PC, Phelps BR, Tolle MA. HIV management by nurse prescribers compared with doctors at a paediatric centre in Gaborone, Botswana. 2013;8.

27. Morrissey B, Kenny T, Williams S, Waddington D. G151(P) Impact of a Training Programme on Non-Medical Health Workers Confidence in Managing Common Neonatal Problems in Sierra Leone. Arch Dis Child. 2013 Jun 1;98(Suppl 1):A70–A70.

28. Hoque DME, Arifeen SE, Rahman M, Chowdhury EK, Haque TM, Begum K, et al. Improving and sustaining quality of child health care through IMCI training and supervision: experience from rural Bangladesh. Health Policy Plan. 2014 Sep 1;29(6):753–62.

29. Nadjm B, Jeffs B, Mtove G, Msuya W, Mndeme L, Mtei F, et al. Inter-observer variation in paediatric clinical signs between different grades of staff examining children admitted to hospital in Tanzania. Trop Med Int Health. 2008 Sep;13(9):1213–9.

30. Kahigwa E, Schellenberg D, Armstrong Schellenberg J, Aponte JJ, Alonso PL, Menendez C. Inter-observer variation in the assessment of clinical signs in sick Tanzanian children. Trans R Soc Trop Med Hyg. 2002 Mar;96(2):162–6.

31. Walter J, Molfino L, Moreno V, Edwards CG, Chissano M, Prieto A, et al. Long-term outcomes of a pediatric HIV treatment program in Maputo, Mozambique: a cohort study. Glob Health Action. 2015 Dec;8(1):26652.

32. Libamba E, Makombe SD, Harries AD, Schouten EJ, Yu JK-L, Pasulani O, et al. Malawi’s contribution to ‘3 by 5’: achievements and challenges. Bull World Health Organ. 2007 Feb;85(2):156–60.

33. Weigel R, Feldacker C, Tweya H, Gondwe C, Chiwoko J, Gumulira J, et al. Managing HIV-infected children in a low-resource, public clinic: a comparison of nurse vs. clinical officer practices in ART refill, calculation of adherence and subsequent appointments. J Int AIDS Soc [Internet]. 2012 Aug 17 [cited 2021 Feb 24];15(2). Available from: http://doi.wiley.com/10.7448/IAS.15.2.17432

34. Rahman AE, Iqbal A, Hoque DME, Moinuddin Md, Zaman SB, Rahman QS, et al. Managing Neonatal and Early Childhood Syndromic Sepsis in Sub-District Hospitals in Resource Poor Settings: Improvement in Quality of Care through Introduction of a Package of Interventions in Rural Bangladesh. Simeoni U, editor. PLOS ONE. 2017 Jan 23;12(1):e0170267.

35. Weigel R, Feldacker C, Tweya H, Chiwoko J, Gumulira J, Phiri S. Nurse-led antiretroviral treatment for HIV infected children: A comparative study from Lilongwe, Malawi. Arch Dis Child. 2012 May;97(Suppl 1):A44.2-A45.

36. Penda CI, Ndongo FA, Bissek A-CZ-K, Téjiokem MC, Sofeu C, Moukoko Eboumbou EC, et al. Practices of Care to HIV-Infected Children: Current Situation in Cameroon. Clin Med Insights Pediatr. 2019 Jan;13:117955651984611.

37. McNairy ML, Bashi JB, Chung H, Wemin L, Lorng M-NA, Brou H, et al. Task-sharing with nurses to enhance access to HIV treatment in Côte d’Ivoire. Trop Med Int Health. 2017 Apr;22(4):431–41.

38. Rhode H, Mash B. The effect of an automated integrated management of childhood illness guideline on the training of professional nurses in the Western Cape, South Africa. South Afr Fam Pract. 2015 Mar 4;57(2):100–5.

39. Nelson SW, Stolz U, Dreifuss BA, Chamberlain S, Hammerstedt HS, Alfunsi B, et al. Training emergency care practitioners and creating access to acute care services in Uganda: The pilot phase. Ann Glob Health. 2014 Sep 25;80(3):172.

40. van Griensven J, De Naeyer L, Uwera J, Asiimwe A, Gazille C, Reid T. Success with antiretroviral treatment for children in Kigali, Rwanda: Experience with health center/nurse-based care. BMC Pediatr. 2008 Dec;8(1):39.

41. Janssen N, Ndirangu J, Newell M-L, Bland R. Successful paediatric HIV treatment in rural primary care in Africa. Arch Dis Child. 2010 Jun 1;95(6):414–21.

42. Patel MR, Yotebieng M, Behets F, Driessche KV, Nana M, Rie AV. Outcomes of integrated treatment for tuberculosis and HIV in children at the primary health care level. :7.

43. Morris MB, Chapula BT, Chi BH, Mwango A, Chi HF, Mwanza J, et al. Use of task-shifting to rapidly scale-up HIV treatment services: experiences from Lusaka, Zambia. BMC Health Serv Res. 2009 Dec;9(1):5.

44. Robertson SK, Manson K, Fioratou E. IMCI and ETAT integration at a primary healthcare facility in Malawi: a human factors approach. BMC Health Serv Res. 2018 Dec;18(1):1014.

45. Sami S, Amsalu R, Dimiti A, Jackson D, Kenyi S, Meyers J, et al. Understanding health systems to improve community and facility level newborn care among displaced populations in South Sudan: a mixed methods case study. BMC Pregnancy Childbirth. 2018 Dec;18(1):325.

46. Hooft AM, Ripp K, Ndenga B, Mutuku F, Vu D, Baltzell K, et al. Principles, practices and knowledge of clinicians when assessing febrile children: a qualitative study in Kenya. Malar J. 2017 Dec;16(1):381.

47. Bessat C, Zonon NA, D’Acremont V. Large-scale implementation of electronic Integrated Management of Childhood Illness (eIMCI) at the primary care level in Burkina Faso: a qualitative study on health worker perception of its medical content, usability and impact on antibiotic prescription and resistance. BMC Public Health. 2019 Dec;19(1):449.

48. Hemming V. G253 Training local staff in northern ethiopia in newborn resuscitation and care. Arch Dis Child. 2015 Apr 1;100(Suppl 3):A109–A109.

49. Chamberlain S, Stolz U, Dreifuss B, Nelson SW, Hammerstedt H, Andinda J, et al. Mortality Related to Acute Illness and Injury in Rural Uganda: Task Shifting to Improve Outcomes. Price MA, editor. PLOS ONE. 2015 Apr 7;10(4):e0122559.

50. James DR, Barling J, Ross O, Daniel AA, Crocker C, Jarvis E, et al. G293 (P) Towards emergency triage assessment and treatment (ETAT)++: introducing basic paediatric trauma management skills in rural ghana. Arch Dis Child. 2019;104(Suppl 2):A120.

51. Rode H, Rogers AD, Numanoglu A, Wallis L, Allgaier R, Laflamme L, et al. A review of primary and secondary burn services in the Western Cape, South Africa. S Afr Med J. 2015 Sep 19;105(10):852.

52. Tyson AF, Msiska N, Kiser M, Samuel JC, Mclean S, Varela C, et al. Delivery of operative pediatric surgical care by physicians and non-physician clinicians in Malawi. Int J Surg Lond Engl. 2014;12(5):509–15.

53. Olayo B, Kirigia CK, Oliwa JN, Agai ON, Morris M, Benckert M, et al. Effective training-of-trainers model for the introduction of continuous positive airway pressure for neonatal and paediatric patients in Kenya. Paediatr Int Child Health. 2019 Jul 3;39(3):193–200.

54. Wilhelm TJ, Thawe IK, Mwatibu B, Mothes H, Post S. Efficacy of major general surgery performed by non-physician clinicians at a central hospital in Malawi. Trop Doct. 2011 Apr;41(2):71–5.

55. Rice B, Periyanayagam U, Chamberlain S, Dreifuss B, Hammerstedt H, Nelson S, et al. Mortality in Children Under Five Receiving Nonphysician Clinician Emergency Care in Uganda. Pediatrics. 2016 Mar;137(3):e20153201.

56. Tindall AJ, Steinlechner CWB, Lavy CBD, Mannion S, Mkandawire N. Results of Manipulation of Idiopathic Clubfoot Deformity in Malawi by Orthopaedic Clinical Officers Using the Ponseti Method: A Realistic Alternative for the Developing World? J Pediatr Orthop. 2005 Sep;25(5):627–9.

57. Wilhelm TJ, Dzimbiri K, Sembereka V, Gumeni M, Bach O, Mothes H. Task-shifting of orthopaedic surgery to non-physician clinicians in Malawi: effective and safe? Trop Doct. 2017 Oct;47(4):294–9.

58. Grimes CE, Mkandawire NC, Billingsley ML, Ngulube C, Cobey JC. The cost-effectiveness of orthopaedic clinical officers in Malawi. Trop Doct. 2014 Jul;44(3):128–34.

59. Tiemeier K, Bisanzo M, Dreifuss B, Ward KC. The Effect of Geography and Demography on Outcomes of Emergency Department Patients in Rural Uganda. Ann Emerg Med. 2013 Oct;62(4):S99.

60. Bowa K, Li MS, Mugisa B, Waters E, Linyama DM, Chi BH, et al. A controlled trial of three methods for neonatal circumcision in Lusaka, Zambia. J Acquir Immune Defic Syndr 1999. 2013 Jan 1;62(1):e1-6.

61. Kankaka EN, Kigozi G, Kayiwa D, Kighoma N, Makumbi F, Murungi T, et al. Efficacy of knowledge and competence-based training of non-physicians in the provision of early infant male circumcision using the Mogen clamp in Rakai, Uganda. BJU Int. 2017 Apr;119(4):631–7.

62. Frajzyngier V, Odingo G, Barone M, Perchal P, Pavin M. Safety of adult medical male circumcision performed by non-physician clinicians in Kenya: a prospective cohort study. Glob Health Sci Pract. 2014 Feb;2(1):93–102.

63. Young MR, Bailey RC, Odoyo-June E, Irwin TE, Obiero W, Ongong’a DO, et al. Safety of over Twelve Hundred Infant Male Circumcisions Using the Mogen Clamp in Kenya. PLOS ONE. 2012;7(10):5.

64. Mwandi Z, Ochieng A, Grund J, Mwalili S, Kimanga D, Otieno G, et al. Service delivery trends in Kenya’s voluntary medical male circumcision scale-up from 2008-2011. In: Journal of the International Aids Society. INT AIDS SOCIETY AVENUE DE FRANCE 23, GENEVA, 1202, SWITZERLAND; 2012. p. 136–7.

65. Al Hussein Alawamlh* Omar, Kim Soo Jeong, Barone Mark, Awori Quentin, Oketch Jairus, Otiende Patrick, et al. Pd08-11 use of topical anesthesia with the shangring male circumcision device: a randomized clinical trial in kenya. J Urol. 2019 Apr 1;201(Supplement 4):e152–3.

66. Kankaka EN, Murungi T, Kigozi G, Makumbi F, Nabukalu D, Watya S, et al. Randomised trial of early infant circumcision performed by clinical officers and registered nurse midwives using the Mogen clamp in Rakai, Uganda. BJU Int. 2017 Jan;119(1):164–70.

67. Aliku TO, Adong C, Kamarembo J, Akech R, Odong F, Apiyo P. PO552 Assessment of the Impact of a Training Program on Knowledge and Clinical Practices of Health Workers Regarding Rheumatic Heart Disease Prevention In Lower Level Health Centers In Gulu Municipality, Uganda. Glob Heart. 2018;13(4):495.

68. Sims AE, Sable CA, Hosseinipour M, Karlsten M, Kazembe PN, Minard CG, et al. Clinical-officer Led Echocardiographic Screening is Sensitive for Diagnosing Rheumatic Heart Disease in Malawi, Africa. Circulation. 2015;132(suppl_3):A18009–A18009.

69. Sanyahumbi A. Education: The prevention of acute rheumatic fever and rheumatic heart disease in Malawi. Malawi Med J. 2019;31(3):221–2.

70. Eberly L, Rusingiza E, Park P, Ngoga G, Dusabeyezu S, Mutabazi F, et al. Heart failure as an entry point for severe cardiovascular disease in sub-saharan Africa: 10-year experience with nurse-led diagnosis and treatment at district hospitals in rural Rwanda. J Am Coll Cardiol. 2018;71(11S):A656–A656.

71. Paiva P, Cappellano A, Dias C, Silva N. Project clinical nurse specialist in neuro-oncology: Experience report. Neuro-Oncol. 2012;14:123–123.

72. Sims Sanyahumbi A, Sable CA, Karlsten M, Hosseinipour MC, Kazembe PN, Minard CG, et al. Task shifting to clinical officer-led echocardiography screening for detecting rheumatic heart disease in Malawi, Africa. Cardiol Young. 2017 Aug;27(6):1133–9.

73. Beaton A, Nascimento B, Diamantino A, Perlman L, Tompsett A, Ribeiro A, et al. PS018 Task-Shifting of Handheld Echocardiographic Screening for Rheumatic Heart Disease: Longitudinal Performance of Non-Experts in a School-Based Program. Glob Heart. 2016 Jun;11(2):e19.

74. Engelman D, Kado J, Remenyi B, Watson C, Rayasidamu S, Steer A, et al. Teaching focused echocardiography for rheumatic heart disease screening. Ann Pediatr Cardiol. 2015;8(2):118.

75. Colquhoun SM, Carapetis JR, Kado JH, Reeves BM, Remenyi B, May W, et al. Pilot study of nurse-led rheumatic heart disease echocardiography screening in Fiji – a novel approach in a resource-poor setting. Cardiol Young. 2013 Aug;23(4):546–52.

76. Ploutz M, Lu JC, Scheel J, Webb C, Ensing GJ, Aliku T, et al. Handheld echocardiographic screening for rheumatic heart disease by non-experts. Heart. 2016 Jan 1;102(1):35–9.

77. Buser JM. The Need for Hematology Nurse Education in Low- and Middle-Income Countries: A Community Case Study in Tanzania. Front Public Health. 2017;5:65.

78. Some D, Edwards JK, Reid T, Van den Bergh R, Kosgei RJ, Wilkinson E, et al. Task Shifting the Management of Non-Communicable Diseases to Nurses in Kibera, Kenya: Does It Work? Griffiths UK, editor. PLOS ONE. 2016 Jan 26;11(1):e0145634.

79. Rossouw J, Yadin E, Alexander D, Mbanga I, Jacobs T, Seedat S. A pilot and feasibility randomised controlled study of Prolonged Exposure Treatment and supportive counselling for post-traumatic stress disorder in adolescents: a third world, task-shifting, community-based sample. Trials. 2016 Nov 17;17(1):548.

80. Harris C, Harris U. Combating barriers to epilepsy treatment in Western Uganda. Trop Med Int Health. 2013 Sep 1;18:83–83.

81. Akol A, Nalugya J, Nshemereirwe S, Babirye JN, Engebretsen IMS. Does child and adolescent mental health in-service training result in equivalent knowledge gain among cadres of non-specialist health workers in Uganda? A pre-test post-test study. Int J Ment Health Syst. 2017;11:50.

82. Patel AA, Wibecan L, Tembo O, Kalyelye P, Mathews M, Ciccone O. Improving paediatric epilepsy management at the first level of care: a pilot education intervention for clinical officers in Zambia. BMJ Open. 2019 Jul;9(7):e029322.

83. Rossouw J, Yadin E, Alexander D, Seedat S. Prolonged exposure therapy and supportive counselling for post-traumatic stress disorder in adolescents: task-shifting randomised controlled trial. Br J Psychiatry J Ment Sci. 2018 Oct;213(4):587–94.

84. Kengne AP, Fezeu LL, Awah PK, Sobngwi E, Dongmo S, Mbanya JC. Nurse-led care for epilepsy at primary level in a rural health district in Cameroon. Epilepsia. 2008 Sep;49(9):1639–42.

85. Kengne AP, Sobngwi E, Fezeu LL, Awah PK, Dongmo S, Mbanya JC, et al. Nurse-Led Care for Asthma at Primary Level in Rural Sub-Saharan Africa: The Experience of Bafut in Cameroon. J Asthma. 2008 Jan;45(6):437–43.

86. Tesfaye M, Abera M, Gruber-Frank C, Frank R. The development of a model of training in child psychiatry for non-physician clinicians in Ethiopia. Child Adolesc Psychiatry Ment Health. 2014 Feb 25;8(1):6.

87. Abbo C, Mwaka AD, Opar BT, Idro R. Qualitative evaluation of the outcomes of care and treatment for children and adolescents with nodding syndrome and other epilepsies in Uganda. Infect Dis Poverty. 2019 Apr 30;8(1):30.

88. van de Water T, Rossouw J, Yadin E, Seedat S. Impediments and catalysts to task-shifting psychotherapeutic interventions for adolescents with PTSD: perspectives of multi-stakeholders. Child Adolesc Psychiatry Ment Health. 2017;11:48.

89. van de Water T, Rossouw J, Yadin E, Seedat S. Adolescent and nurse perspectives of psychotherapeutic interventions for PTSD delivered through task-shifting in a low resource setting. PloS One. 2018;13(7):e0199816.

90. Mafwiri MM, Kisenge R, Gilbert CE. A pilot study to evaluate incorporating eye care for children into reproductive and child health services in Dar-es-Salaam, Tanzania: a historical comparison study. BMC Nurs. 2014 Dec;13(1):15.
