# Supplementary material for: Task-sharing to support paediatric and child health service delivery in low- and middle-income countries: current practice and a scoping review of emerging opportunities
Source: Hum Resour Health. 2021 Aug 4;19:95. doi: 10.1186/s12960-021-00637-5 (PMC8336272; doi:10.1186/s12960-021-00637-5)
Supplement: Supplementary file 2 — Additional file 2: Training opportunities and scope of practices related to child health for mid-level health workers in Kenya, Uganda, Tanzania, Malawi and South Africa. [file 12960_2021_637_MOESM2_ESM.docx]

## Appendix 2. Training opportunities and scope of practices related to child health for mid-level health workers in Kenya, Uganda, Tanzania, Malawi and South Africa

Note: for nurses we only looked at their post-basic training opportunities excluding diploma and bachelor’s degree, despite that bachelor’s degrees could also be post-basic degree for diploma or certificate holders

|  |  | **Training related to child health** | **Other specialized training** | **Scope of practice, relevant national policy and planning** |
| --- | --- | --- | --- | --- |
| **Kenya** | **Medical officer** | - Bachelor of Medicine & Bachelor of Surgery (MBChB) (6 years, direct entry) that includes courses and junior/senior clerkship in child health and paediatrics and 3-month internship in paediatrics - Master of Medicine in Paediatrics and Child Health or Paediatric Surgery and paediatrics sub-speciality under anaesthesia, pathology, cardiac surgery, emergency medicine, otolaryngology, etc; (3-4 years for speciality, 6 months for subspeciality, after the MBChB degree and some also requires one-year work experience) that cover major body systems and diseases - Fellowship in Paediatrics anaesthesia, neurology, neonatology, etc. (2 years, after MMed and 4 year post internship experience) - Continuous Professional Development (CPD) courses | - Master of Medicine in Family Medicine, Anaesthesia, Pathology, Cardiothoracic and Vascular Surgery, Dermatology, Emergency Medicine, ENT Surgery, General Surgery, Immunology, Internal Medicine, Medical Microbiology, Neurology, Obstetrics and Gynaecology, Occupational Medicine, Oncology, Ophthalmology, Orthopaedic Surgery, Palliative Medicine, Plastic and Reconstructive Surgery, Psychiatry, Urology, Radiology, Public Health (4-7 years, after the MBChB degree and some also requires two-year work experience) - Fellowship in in cardiology, infectious diseases. (2 years, after MMed and 4 year post internship experience | - The Scheme of Service (2016) document indicates that medical officers’ duties and responsibilities include diagnosing, caring and treating diseases; performing medical and surgical procedures; preparing and responding to emergencies and disasters; management of medicines and equipment’ maintaining health information and data; counselling; teaching medical students, nursing students and clinical officer interns; senior medical officers’ also include clerking, investigating and managing patients; conducting weekly clinics and theatre days; supervising medical officer interns and other students etc. No paediatric-specific services are defined. Medical specialists I and above can carry out specialized clinical care aside from general diagnosis, care, treatment and rehabilitation. - The Medical Practitioners and Dentists Act (2019) does not specify any services that are allowed or not allowed - The Medical Practitioners and Dentists (Private Medical Institutions) (Amendment) Rules (2017) and the Human Resources for Health Norms and Standards Guideline (2014) requires at least two medical officers in level 3 comprehensive health centres, at least sixteen medical officers and two paediatricians in level 4 hospitals and more numbers required at level 5 and 6 hospitals - The Task Sharing Guideline/Policy (2017) lists tasks that could be shared down to lower cadres (see below) - The National Guidelines and Logbook for Medical Officer Interns (2019) lists out the conditions for paediatric emergencies, chronic conditions and practical skills that medical officers need to show competency in. |
|  | **Clinical officer** | - Diploma in Clinical Medicine and Surgery (3 years, direct entry) that includes 4 paediatrics and child health course, 3-month rotation in paediatrics and child health - Bachelor of Science in Clinical Medicine and Community Health (4 years, direct entry or after the diploma) that includes one child health course, 3-month rotation in paediatrics and child health - Higher Diploma in Paediatrics (1 years, after the diploma or bachelor’s degree + 3 years working experience) that covers major subspecialities including neonatology, forensic medicine, haematology, surgery, nutrition and endocrinology, mental health etc. - Continuous Professional Development (CPD) courses | - Higher Diploma in Family Medicine, Ophthalmology and Cataract Surgery, Dermatology and Venerology, Chest Medicine, ENT and Neck Surgery, Anaesthesia, Mental Health and Psychiatry, Ophthalmology Advanced Refraction and Low Vision, Oncology and Palliative Care, Audiology and Hearing Care Technology, Emergency and Critical Care Medicine, Orthopaedics, General Ophthalmology (1.5 years, after the diploma or bachelor’s degree + 3 years working experience) - Master in Clinical Medicine in Forensic Medicine, Accident and Emergency Medicine, Family Health (3 years, after the bachelor’s degree) no paediatrics option yet but under curriculum development | - The Scheme of Service (2014) document only indicates that clinical officers could offer specialized services in paediatrics and child health including history taking, examining, diagnosing, treating and managing diseases and conditions in outpatient or inpatient facilities, carrying out minor surgical procedures per training and skills. Higher grade clinical officers can carry out surgical procedure per training and skills and offer specialized clinical services, as well as coach/mentor students and conduct ward rounds - The Clinical Officer Act (2012) and Clinical Officer Bill (2016) lists out activities that clinical officers licensed to engage in private practice are permitted to treat, which are broad terms on “advice on general care for newborn”, “gastrointestinal conditions”, “poisoning”, “burns”, etc.; additionally, a list of drugs and equipment are allowed to be prescribed by clinical officers - The Task Sharing Guideline/Policy (2017) lists tasks that may be conducted with adequate training and supervision, which include essential newborn care, child and adolescent care that focus on counselling, nutrition and development at both primary level (level 1-2 as the highest cadre) and higher level (level 3-5). Prescription is only mentioned for HIV/AIDS and tuberculosis conditions. - The Human Resources for Health Norms and Standards Guideline (2014) indicates that for level 1 (community units) and 2 (dispensary) clinical officers are the key cadre providing primary care and treatment, preventive and promotive care; for level 3 (health centre) there should be 2 medical officers, 11 clinical officers including one paediatrics clinical officers |
|  | **Nurse** | - Higher Diploma in Paediatric Nursing/Neonatal Nursing/Paediatric Critical Care Nursing/Neonatal Critical Care Nursing (1 year, after diploma or bachelor’s degree + 2 years working experience) - Master of Science in Nursing Science in Paediatric Nursing (2 years, after bachelor’s degree + 2 years working experience) - Continuous Professional Development (CPD) courses | - Higher Diploma in Family Health Nursing, Critical Care Nursing, Psychiatric Nursing, Palliative Care, Nephrology Nursing, Oncology Nursing, Peri-Operative Nursing, Accident and Emergency Nursing (1-1.5 years, after diploma or bachelor’s degree + 2 years working experience) - Short courses offered on Stoma and Wound Care (3 months), Basic Life Support, Advanced Cardiac Life Support, Advanced Trauma Life Support | - The Scheme of Service (2014) document lists that entry level nurse could provide appropriate healthcare services including immunization, PMTCT, etc. Additionally, higher grade nurses could provide IMCI, Integrated Management of Adult and Adolescent Illness, and teaching nursing staff and students - The Nurse Act (2016) does not specify any services that are allowed or not allowed - The Task Sharing Guideline/Policy (2017) same with clinical officers, including essential newborn care, child and adolescent care that focus on counselling, nutrition and development at both primary level (level 1-2) and higher level (level 3-5) - The Human Resources for Health Norms and Standards Guideline (2014) indicates paediatrics nurses only required at level 4 (county hospital) and above |
| **Uganda** | **Medical officer** | - Bachelor of Medicine and Bachelor of Surgery (MBChB) (5 years, direct entry) and one-year internship including in paediatrics - Master of Medicine in Paediatrics and Child Health (2 or 3 years, after bachelor’s degree and 2 years working experience) - Paediatrics Surgery, Paediatric Cardiology fellowship (3 years, after MMed degree) | - Master of Medicine in General Surgery, Internal Medicine, Dermatology, Anaesthesia, Ophthalmology, Obstetrics and Gynaecology, Community Practice/Family Medicine, ENT, Emergency Medicine, Radiology, Pathology, Psychiatry, Plastic and Reconstructive Surgery (2 or 3 years, after bachelor’s degree and 2 years working experience) - Fellowship in Neurosurgery, Cardiology, etc. (3 years, after MMed) | - The Medical and Dental Practitioners Act (1998) does not specify any services that are allowed or not allowed - The Health Service Commission Guidelines for the Recruitment of Health Workers in Local Government (2020) lists for medical officers, key functions include participating in planning and budget activities; diagnosing, treating and referring patients; accounting for allocated resources; participating in outreach health programs; research activities; continued professional development; adhering to professional code of conduct and ethics; compiling and submitting reports; for senior medical officers (general hospital) additional functions include supervising, coaching, mentoring and appraising staff; for senior medical officer (health centre IV or >3 years at MO), additional functions include managing health information management systems; coordinating the procurement of equipment, drugs and other supplies; coordinating the implementation of Uganda National Minimum Health Care Package; providing guidelines and enforcing adherence to standards; providing technical guidance and supervision to health centres; overseeing the operationalization of the Occupational Health and Safety Policy and Guidelines; ensuring regular maintenance and functioning of the equipment at the Health Centre; and supervising, coaching, mentoring and appraising staff; for medical officer special grade (pediatrics and child health) additional functions include planning and budgeting for Paediatric programs; and supervising, coaching, mentoring and appraising staff - The Staffing Norm under Health Service Commission Guidelines for the Recruitment of Health Workers in Local Government (2020) indicates that Health Centre IV requires at least one medical officer and one senior medical officer; and general hospital requires at least one principal medical officer, five special grade medical officer (one in pediatrics and child health), one senior medical officer and 3 medical officers - The Child and Adolescent Mental Health Policy (2017) recommends improving training of nursing staff, social workers, counsellors, psychologists, clinical officers, occupational therapists, medical officers, paediatricians, psychiatrists in the detection, diagnosis, and management of children and adolescents suffering from mental, neurological and substance use disorders |
|  | **Clinical officer** | - Diploma in Clinical Medicine and Community Health (3 years, direct entry) that include one public health course on maternal and child health and two paediatrics courses, as well as clinical attachment and internship - Bachelor of Science in Clinical Medicine and Community Health (4.5 years, direct entry or after the diploma) including two courses on child health and paediatrics and internship - Advanced diploma in Child and Adolescent Mental Health, Paediatric Palliative Care, and Child Counselling (2 years, after diploma or bachelor’s degree with working experience) | - Advanced Diploma in Anaesthesia, ENT and Neck Surgery, Speech and Language Therapy (1.5-2 years, after diploma) | - The Allied Health Professional Act (1996) does not specify any services that are allowed or not allowed - The Health Service Commission Guidelines for the Recruitment of Health Workers in Local Government (2020) lists for Clinical officers, key functions include diagnosing and treating; referring patients; participating in continuous professional development activities; research activities; conduction health education to patients; adhering to the code of conduct and ethics; accounting for allocated resources; compiling and submitting reports; for senior clinical officer (general hospital), additional functions include diagnosing, treating and managing patients; supervising, coaching, mentoring and appraising staff   for senior clinical officer (HC III in charge), additional functions include planning and budgeting for health services delivery activities in the Health Centre; diagnosing, treating and managing patients; managing and accounting for allocated medical, fiscal and other resources; supervising health management information system; maintenance of the unit infrastructure and equipment; requisitioning the procurement of equipment, medicines and other supplies for the Health Centre; supervising, coaching, mentoring and appraising staff; coordinating the implementation of the Uganda National Minimum Health Care Package; providing technical guidance and supervision to Health Centre II; implementing continuous Professional Development programs; reports compiled and submitted.   - The Staffing Norm under Health Service Commission Guidelines for the Recruitment of Health Workers in Local Government (2020) indicates that indicates that Health Centre III requires at least one senior clinical officer and one clinical officer; Health Centre IV requires at least two clinical officer; and general hospitals at leats one senior clinical officer, one psychiatrics and one ophthalmic clinical officer and five clinical officers - The Child and Adolescent Mental Health Policy (2017) recommends improving training of nursing staff, social workers, counsellors, psychologists, clinical officers, occupational therapists, medical officers, paediatricians, psychiatrists in the detection, diagnosis, and management of children and adolescents suffering from mental, neurological and substance use disorders |
|  | **Nurse** | - Bachelor of Science in Nursing (BSN) (4 years, direct entry or extension) plus one year internship including in paediatrics - Diploma of Nursing (extension) (2 years, after certificate and 2 years working experience) or Advanced Diploma in Paediatric and Child Health Nursing (1.5 years, registered with nursing council and 2 years working experience) that covers most sub-specialties including respiratory, cardiovascular, neurological, urinary endocrine, skin, eye conditions, HIV and IMCI | - Diploma in Mental Health Nursing (direct or extension) (1.5 years for extension) - Advanced Diploma in Public Health Nursing, Palliative Care (1 year, after diploma, some require 2 year working experience) - Master of Nursing Sciences (Multiple Track including women’s, and midwifery) (2 years, after bachelor’s degree) focusing on critical care of both adult and child | - The Scheme of Service (2017) document is broad and lists nurses provide counselling, therapeutic care (institute prescriptions, provide nursing care, maternal and child care, documentation) as we as other functions. Higher grade nurses can also coach and mentor enrolled nurses - The Nursing Act (1996) defines nurses as “a person who is trained and qualified in the promotion of health, the prevention of diseases and the care of the sick and who is registered or enrolled under section 23” - The Health Service Commission Guidelines for the Recruitment of Health Workers in Local Government (2020) lists for enrolled nurses, key functions include participating in continuous coverage on wards and units; administering treatment as prescribed; carrying out nursing procedures; carrying out observations, keep proper records and ensure their safe custody; participating in ward rounds; receiving and registering patients; preparing patients for meals and participate in serving them; adhering to aseptic procedures; adhering to ethical professional conduct; carrying out health education; participating in primary health care activities; for senior enrolled nurse, additional functions include providing quality nursing services; assessing patients and clients; providing a therapeutic and comfortable environment; monitoring patients and clients condition, and keep proper records; for assistant nursing officer (nursing), additional functions include preparing and submit report; allocating and supervising duties of Enrolled Nurses; coaching and mentoring Enrolled Nurses; implementing nursing protocols; managing performance of Enrolled Nurses; accounting for supplies and drugs; for nursing officer (nursing) additional functions include planning, monitoring, and evaluating nursing activities; allocating duties to nursing staff and students for smooth running of wards of heath units; coaching and mentoring students and staff; managing performance of staff; managing wards and providing adequate supplies and drugs; for senior assistant nursing officer (nursing) additional functions include providing support supervision in nursing care; preparing and implementing Nursing services; maintaining a clean and healthy ward environment; for senior nursing officer (nursing) additional functions include planning, monitor, and evaluate nursing activities; managing performance of staff; supervising, coaching, and mentoring staff and students etc. - The Staffing Norm under Health Service Commission Guidelines for the Recruitment of Health Workers in Local Government (2020) indicates that indicates that indicates that Health Centre II requires at least one enrolled nurse, one enrolled midwife, one health assistant and one nursing assistant; Health Centre III requires at least three enrolled nurses, two enrolled midwives, one nursing assistant; Health Centre IV requires at least three enrolled nurses, two enrolled midwives and five nursing assistants; general hospitals requires at least one principal assistant nursing officer, five senior assistant nursing officer; 21 assistant nursing officer (17 in nursing)l two enrolled psychiatric nurse; 46 enrolled nurse and 25 enrolled midwives - The Child and Adolescent Mental Health Policy (2017) recommends the same for nurses as clinical officers |
| **Tanzania** | **Medical officer** | - Doctor of Medicine (Bachelor) (5 years, direct entry or after diploma) that includes one-year internship - Master of Medicine in Paediatric and Child Health (3-4 years, after MD degree and one year working experience) - Master of Science in Paediatric Haematology, and Paediatric Surgery (super-specialization) (2 years, after MMed degree) | - Master of Medicine in Family Medicine, Anaesthesia, Dermato-venereology, Diagnostics Radiology, Emergency Medicine, General Surgery, Internal Medicine, Obstetrics and Gynaecology, Orthopaedics and Traumatology, Ophthalmology, Otorhinolaryngology, Pathology, Psychiatry and Mental Health, Urology, etc. (3-4 years, after MD degree and one year working experience) - Master of Science in Cardiology, Haematology and Blood Transfusion, Nephrology, Neurology, Respiratory Medicine, Medical Gastroenterology and Hepatology, Surgical Gastroenterology and Hepatology, Neurosurgery, Urology (super-specialization) (2 years, after MMed degree) | - The Scheme of Service (2009) document lists for medical officer could perform all hospital duties including paediatric, gynaecological, routine and emergency surgery, train and supervise students and staff, etc. Medical specialists and consultants are allowed to perform duties in their relevant field - The Medical Dental and Allied Health Professional Act (2017) does not specify any services that are allowed or not allowed - Task sharing policy guideline (2016) requires medical officers at health centre level and above, and the tasks that could be shifted to other cadres are described below - The Staffing Levels for Ministry of Health and Social Welfare Departments, Health Service Facilities, Health Training Institutions and Agencies (2014) indicates for health centres (first level referral outpatient and inpatient) should have 1 medical officer, district hospitals should have 8-23 medical officers (1-4 for paediatric ward), regional referral hospitals should have 20-30 medical officers (2 for paediatric clinic, 1-5 for paediatric ward) and 21-24 specialists (2 for paediatric clinic, 1-3 for paediatric ward) |
|  | **Assistant medical officer** | - Advanced Diploma in Clinical Medicine (2 years, after ordinary diploma [some mentioned 3 years working experience required]) that includes rotation in paediatrics (no longer offered) | - Advanced Diploma in Dermatovenerology (2 years, after ordinary diploma) - Advanced Diploma in Anaesthesia, Radiology, Ophthalmology (2 years) | - The Scheme of Service (2009) document specifically lists assistant medical officers could provide treatment, prevention and care for children, perform emergency and routine surgery, higher grade assistant medical officers could also supervise subordinates - The Medical Dental and Allied Health Professional Act (2017) does not specify any services that are allowed or not allowed - Task sharing policy guideline (2016) indicates task sharing for assistant medical officers happen at health centre and district hospital level primarily and less in referral hospitals. Tasks include nutrition (treatment of severe malnutrition, micronutrient supplementation), uncomplicated acute respiratory infections (IMCI), diarrhoea, general prescribing medication, surgery, ward rounds, counselling, and supervision, etc. - The Staffing Levels for Ministry of Health and Social Welfare Departments, Health Service Facilities, Health Training Institutions and Agencies (2014) indicates for health centres (first level referral outpatient and inpatient) should have 1 medical officer and 1 assistant medical officer covering both outpatient and inpatient; no requirement for assistant medical officers in dispensary (first-level primary health care outpatient) |
|  | **Clinical officer** | - Ordinary Diploma in Clinical Medicine (3 years for direct entry or 1 year for certificate holder) that includes four courses on paediatrics and child health | - N/A | - The Scheme of Service (2009) document is broad and lists clinical officers could diagnosis and treat common disease, perform minor surgery, as well as supervise subordinates - The Medical Dental and Allied Health Professional Act (2017) does not specify any services that are allowed or not allowed - Task sharing policy guideline (2016) indicates task sharing for clinical officer/clinical assistant happen at dispensary and health centre and not in district hospitals. Tasks include nutrition (treatment of severe malnutrition, micronutrient supplementation), uncomplicated acute respiratory infections (IMCI), diarrhoea, general prescribing medication (at dispensary), minor surgery (incision and drainage at dispensary), and supervision, etc. - The Staffing Levels for Ministry of Health and Social Welfare Departments, Health Service Facilities, Health Training Institutions and Agencies (2014) indicates dispensary (first-level primary health care outpatient) should have 1-2 clinical officer/clinical assistants as the highest cadre, covering outpatient and emergency; for district hospital there is no requirement for clinical officers/assistants |
|  | **Clinical assistant / assistant clinical officer** | - Certificate in Clinical Medicine (2 years, direct entry) that is the first two years of the ordinary diploma | - N/A | - The Scheme of Service (2009) document is broad and lists could provide prevention, diagnosis and treatment of diseases and provide primary health care - The Medical Dental and Allied Health Professional Act (2017) does not specify any services that are allowed or not allowed - Task sharing policy guideline (2016) does not differentiate clinical assistant with clinical officer - The Staffing Levels for Ministry of Health and Social Welfare Departments, Health Service Facilities, Health Training Institutions and Agencies (2014) indicates dispensary (first-level primary health care outpatient) should have 1-2 clinical officer/clinical assistants as the highest cadre, covering outpatient and emergency; for district hospital there is no requirement for clinical officers/assistants |
|  | **Nurse** | - Advanced Diploma in Paediatric Nursing (2 years, after diploma and 2 years working experience) - Master of Sciences in Paediatric Nursing (2 years, after bachelor’s degree and 2 years working experience) | - Advanced Diploma in ophthalmic nursing, operating theatre management, psychiatric nursing, health education (2 years, after diploma) - Master of Sciences in Critical Care and Trauma Nursing, Nursing Mental Health (2 years, after bachelor’s degree and 2 years working experience) | - The Scheme of Service (2009) document is broad and lists could provide nursing, preventive and reproductive services. Higher grade nurses could also supervise subordinates - The Nursing and Midwifery Act (2017) does not specify any services that are allowed or not allowed - Task sharing policy guideline (2016) indicates task sharing for clinical officer/clinical assistant happen at dispensary, health centre and district hospital. Tasks that could be shared are the same with clinical officer/assistant (at dispensary, health centre) and assistant medical officer (district hospital), other than nurses cannot treat severe malnutrition at health centre level |
|  | **Medical attendant** | - Certificate in nursing, community health or related field (1 year, direct entry) | - N/A | - The Scheme of Service (2009) document is broad and scope mostly supportive (cleaning, feeding, etc.) - Task sharing policy guideline (2016) indicates task sharing for clinical officer/clinical assistant happen at dispensary, health centre and district hospital. In dispensary, medical attendants can perform minor surgery (incision, drainage), prescribe medication; at health centre level manage uncomplicated acute respiratory infection (IMCI); at district level mostly supportive role. - The Health Sector Strategic Plan (July 2015 – June 2020) intends to formalise current untrained Ward Attendants to skilled Health Attendants, by taking them through competency-based training - The Staffing Levels for Ministry of Health and Social Welfare Departments, Health Service Facilities, Health Training Institutions and Agencies (2014) lists medical attendant’s function as “general cleanness” |
| **Malawi** | **Doctor** | - Bachelor of Medicine, Bachelor of Surgery (MBBS) (5 years, direct entry) that includes 18-month internship (4 months in paediatrics) - Master of Medicine in Paediatrics and Child Health (4 years, after bachelor’s degree and 1-2 year working experience) - Continuous Professional Development (CPD) courses | - Master of Medicine in Family Medicine, Medicine, Anaesthesia, Ophthalmology, Emergency Medicine, Obstetrics and Gynaecology, Otolaryngology, Psychiatry, Orthopaedics, Surgery (4 years, after bachelor’s degree and 1-2 year working experience) | - Medical Practitioners and Dentists Act (1987) does not specify any services that are allowed or not allowed - The Guideline for Internship Training (2002) is broad and lists intern should gain experience in management of medical and surgical emergencies and, where feasible, to perform those procedures himself or herself under supervision. There is no paediatric specific condition listed - The Health Sector Strategic Plan (2017-2022) lists the minimum staff norms (one medical person (doctor, clinical officer or medical assistant), two Nurse-Midwives, one medical technician, one Environmental Health Officer, two hospital attendants) to offer essential health package services |
|  | **Clinical officer** | - Diploma in Clinical Medicine (3 years for direct entry, 1.5 years for certificate holder with 2 years working experience) that also includes one-year internship - Bachelor of Science in Paediatrics and Child Health (3 years, after diploma and 2 years working experience) that focus on district and sub-district level | - Bachelor of Science in Internal Medicine, Obstetrics and Gynaecology, General Surgery, Anaesthesia and Intensive Care (3 years, after diploma and 2 years working experience) that focus on district and sub-district level - Postgraduate diploma in HIV Medicine (1.5 years, after BSc degree) | - Medical Practitioners and Dentists Act (1987) does not specify any services that are allowed or not allowed - The Human Resource for Health Strategic Plan (2012-2016) acknowledges clinical officers, medical assistants and/or registered nurses or nurse/midwife technician (NMTs) are responsible for delivering most district health services that would normally be provided by fully qualified doctors or specialists - The Health Sector Strategic Plan (2017-2022) lists the minimum staff norms (one medical person (doctor, clinical officer or medical assistant), two Nurse-Midwives, one medical technician, one Environmental Health Officer, two hospital attendants) to offer essential health package services |
|  | **Medical assistant** | - Certificate in Clinical Medicine (2 years, direct entry) without internship requirement | - N/A | - Medical Practitioners and Dentists Act (1987) does not specify any services that are allowed or not allowed - The Human Resource for Health Strategic Plan (2012-2016) acknowledges similar role of medical assistants delivering district health services with clinical officers and nurses - The Health Sector Strategic Plan (2017-2022) lists the minimum staff norms and medical assistants are similar with clinical officers when offering essential health package services |
|  | **Nurse** | - Bachelor of Science in Child Paediatric Nursing (post-basic) (4 years for direct entry, 2 years for mature entry i.e. registered nurses with 2 years working experience) - Master of Science in Child Health Nursing (2 years, after bachelor’s degree and 2 years working experience) - Continuous Professional Development (CPD) courses | - Bachelor of Science in Adult Health Nursing, Community Health Nursing (post-basic) (4 years for direct entry, 2 years for mature entry i.e. registered nurses with 2 years working experience) - Postgraduate diploma in HIV Medicine (1.5 years, after BSc degree) - Master of Science in Adult Health Nursing, Community health nursing, Reproductive Health Nursing (2 years, after bachelor’s degree and 2 years working experience) | - Nursing and Midwifery Policy (2018) lists nurse’ scope of practice include medical-surgical nursing (adults and children); mental health/psychiatric nursing; sexual, reproductive, maternal and neonatal health services (SRMNH) and adolescent-friendly services, in both hospital and community settings - Professional Practice Standards for Registered Nurses (2012) document is broad. Specifically, nurses can manage (including prescribing treatment) common or minor primary health conditions presented at primary care facilities in accordance with the country's norms and standards and standard treatment guidelines. - Nursing and Midwifery Act (1966) does not specify any services that are allowed or not allowed - The Human Resource for Health Strategic Plan (2012-2016) acknowledges similar role of nurses delivering district health services with clinical officers and medical assistants - The Health Sector Strategic Plan (2017-2022) lists the minimum staff norms (one medical person (doctor, clinical officer or medical assistant), two Nurse-Midwives, one medical technician, one Environmental Health Officer, two hospital attendants) to offer essential health package services |
| **South Africa** | **Doctor** | - Bachelor of Medicine and Bachelor of Surgery (MBChB) (5-6 years for direct entry or 4 years for graduate entry) that includes courses in child health and paediatrics, 4-month internship in paediatrics and one year community service - Diploma in Child Health (self-study exam, after MBChB degree) - Postgraduate Diploma in Community and General Paediatrics (2 years, part time after MBChB degree) and Postgraduate Diploma in sub-specialities including, Ambulatory and Emergency Service, Paediatric Anaesthesia, Paediatric Cardiology, Paediatric Critical Care, Development Paediatrics, Paediatric Endocrinology, Paediatric Gastroenterology, Paediatric Haematology/Oncology, Neonatology, Paediatric Nephrology, Paediatric Neurology, Paediatric Physiotherapy, Paediatric Pulmonology, Paediatric Rheumatology, Paediatric Surgery (1-2 year, after MBChB degree) - Registrar training or Master of Medicine in Paediatrics (4 years, after MBChB degree and some work experience) - Master of Philosophy in Paediatrics sub-specialities including Paediatric Allergology, Paediatric Anaesthesia, Child and Adolescent Psychiatry, Paediatric Cardiology, Paediatric Critical Care, Development Paediatrics, Paediatric Endocrinology, Paediatric Gastroenterology, Neonatology, Infectious Diseases, Paediatric Nephrology, Paediatric Neurology, Paediatric Physiotherapy, Paediatric Pulmonology, Paediatric Rheumatology, Paediatric Surgery (2-5 years, after MBChB degree and requires holding senior registrar posts) | - Diploma in Obstetrics, Anaesthesiology, Primary Emergency Care, Ophthalmology, HIV Management, Internal Medicine, Orthopaedics (self-study exam, after MBChB degree) - Postgraduate Diploma in Family Medicine, Palliative Medicine, etc. (1 year full time or 2 years part time after MBChB degree - Registrar training or Master of Medicine in Anaesthesia, Anatomical Pathology, Cardio-Thoracic Surgery, Chemical Pathology, Clinical Pathology, Community Health, Dermatology, Diagnostic Radiology, Forensic Pathology, Haematology, Internal Medicine, Neurology, Neurosurgery, Nuclear Medicine, Microbiology, Obstetrics & Gynaecology, Ophthalmology, Orthopaedic Surgery, Otorhinolaryngology, Plastic and Reconstructive Surgery, Psychiatry, Radiation Oncology, Surgery, Urology, Virology (4 years, after MBChB degree and some work experience) | - Health Professional Act (1974) Regulations defining the scope of practice of medicine is broad and lists out that medical professions can provide physical medical and clinical exams, perform medical and clinical procedure, prescribe medicine, substance or medical devices, and other act based on their education and training as approved by the board. - The Health Professional Act (1974) Regulations specialities and subspecialities in medicine and dentistry (2001) and amendment (2020) require that to register as a specialist, a person need four years’ education and training in board approved post as registrar - No publicly-availably task sharing policy - The Strategic Plan for Maternal, Newborn, Child and Women’s Health and Nutrition (2012-2016) mentioned that a district clinical specialist teams will be made up of an obstetrician, a paediatrician, a family physician, an anaesthetist, an advanced midwife, an advanced paediatric nurse and a PHC nurse - The Medical and Dental Professions board Levels of Health Care (Human Resources, Services, Levels of Care, Equipment and Drugs) (2011) requires medical officers (full time and part-time) and some senior medical officers at community health centre, more medical officers (one available on call at night) for district hospitals, and specialists in regional hospitals. - The Handbook on Internship Training: Guidelines for Interns, Accredited Facilities and Health Authorities (2017) lists out the 16 specific procedures to be performed, 9 inpatient protocols (asthma, diabetic ketoacidosis, cardiac failure, etc.), 10 outpatient (developmental delay, otitis media, anaemia, etc.) and 5 casualties (epilepsy, drowning, etc). |
|  | **Clinical associate** | - Bachelor of Clinical Medical Practice (3 years, direct entry) that includes child health courses | - Bachelor of Clinical Medical Practice Honours in Emergency Medicine (2 years part time, after the BCMP degree) | - Health Professional Act (1974) Regulations defining the scope of practice of clinical associates (2016) lists out that clinical associates can obtain patient history, order and/or perform diagnostic and therapeutic for common and important conditions, perform and/or order selected investigations, interpret and formulate diagnosis for common and emergency conditions, perform selected procedures under registered medical practitioner supervision (for children under 12 there are 12 procedure allowed including lumbar puncture [not for neonate], hearing screen, immunization, IMCI, nutrition assessment, gallows traction, intraosseous access, neonatal and paediatrics resuscitation, initiate CPAP in RDS, photography; for 13-17 there are ~80 procedures), prescribe medicine for common and important conditions under primary care level essential drug list and schedule IV except in emergencies when appropriate drugs may be subscribed, being assistant at surgery, make appropriate admission, discharges and referral, assist medical practitioners within district level health care services and with the focus on primary health care.. They shall not conduct independent private practice and must be under continuous supervision of medical practitioner within first two years; after five years they can practice independently and only need to report when necessary. - No publicly-availably task sharing policy - The 2030 Human Resource for Health Strategy: Investing in the Health Workforce for Universal Health Coverage and Strategic Plan 2020/21-2024/25 (2020) mentioned the need for policy decisions on the scaling up of mid-level workers, other task-shifting or task sharing interventions, and the introduction of new nursing categories, it also mentioned there is no national determination of appropriate staffing ratios or facility staffing norms - General Regulations of the Medicine and Related Substances Act (1965) did not include clinical associates to prescribe and therefore clinical associates are not allowed to prescribe - The Official Statement concerning the Dispensing of Clinical Associates prescriptions by Pharmacists (2010) confirmed that clinical associates have not been approved as authorises prescribers. - The National Drug Policy (1996) mentioned at primary level prescribing will be competency, not occupation, based. |
|  | **Nurse and nurse specialist** | - Postgraduate/Advanced Diploma in Child Health (community paediatrics and neurodevelopment), or Child Nursing, or Critical Care Child Nursing, or Advanced Midwifery and Neonatal Care (1 year, after four year BSc degree or diploma, 2 years working experience preferrable) - Master of Science/Nursing in Child Nursing, or Neonatal Nursing, or Advanced Midwifery and Neonatal Care (2 years, after BSc nursing degree) | - Postgraduate/Advanced Diploma in Primary Health Care, Nephrology Nursing, Neuroscience Nursing, Ophthalmic Nursing (1 year, after four year BSc degree or diploma, 1-2 years working experience preferrable) - Master of Science/Nursing in Advanced Psychiatric Nursing, Infection Control, Intensive Care, Nephrology, Nursing Education, Occupational Health Nursing, Oncology and Palliative Care, Trauma and Emergency Nursing (2 years, after BSc nursing degree) | - Nursing Act (2005) Regulations regarding scope of practice for nurses and midwives lists out that professional nurses can provide comprehensive nursing treatment and care, manage nursing care, provide emergency care, ensure safe implementation of nursing care and administration of medication prescribed by an authorized registered person, etc. Professional nurses may not act as assistant to surgeon or medical practitioners during surgical procedures. - The South Africa Nursing Council lists out different competencies for advanced practice nurses including generic competency, critical nurse specialist, nephrology nurse specialist, occupational health nurse specialist, ophthalmic nurse specialist, paediatrics nurse specialist, peri-operative nurse specialist and primary care nurse specialist. For paediatrics nurse specialist, its competency under domain 2 (clinical practice) include health teaching and promotion, assessment, diagnosis (including “Formulates a diagnosis, prioritising emergences and life-threatening conditions”, “Utilises expert knowledge to interpret results of screenings and diagnostic investigations performed”) planning (“Develops a prioritised plan of care that includes interventions and alternatives to attain expected outcomes for the child ”), implementation, evaluation and therapeutic communication and relationship. - No publicly-availably task sharing policy - The 2030 Human Resource for Health Strategy: Investing in the Health Workforce for Universal Health Coverage and Strategic Plan 2020/21-2024/25 (2020) mentioned the need for policy decisions on the scaling up of mid-level workers, other task-shifting or task sharing interventions, and the introduction of new nursing categories, it also mentioned there is no national determination of appropriate staffing ratios or facility staffing norms - The National Drug Policy (1996) mentioned at primary level prescribing will be competency, not occupation, based - The Strategic Plan for Maternal, Newborn, Child and Women’s Health and Nutrition (2012-2016) mentioned that a district clinical specialist teams will be made up of an obstetrician, a paediatrician, a family physician, an anaesthetist, an advanced midwife, an advanced paediatric nurse and a PHC nurse; it also mentioned that with ART guidelines and NIMART (nurse-initiated management of antiretroviral therapy) nurses are allowed to provide ART at all primary care facility level |
